# Enabling Scalable Real-Time Sensor Stream Processing Across Decentralized Privacy-Preserving Storage Solutions

**DOI:** 10.3390/s26185846

**Published:** 2026-09-15

**Authors:** Kushagra Singh Bisen, Stijn Verstichel, Femke Ongenae

**Affiliations:** IDLab, iGent Tower—Department of Information Technology, Ghent University—imec, Technologiepark-Zwijnaarde 126, B-9052 Ghent, Belgium; stijn.verstichel@ugent.be (S.V.); femke.ongenae@ugent.be (F.O.)

**Keywords:** sensor stream processing, decentralization, personal data processing, solid

## Abstract

Internet of Things (IoT), Smart Health, and Smart City applications generate continuous data streams that may contain sensitive personal information. Decentralized storage systems such as Solid provide data ownership, access control, and interoperable sharing but offer limited support for real-time stream processing. We present Heimdall, an intermediary analytics service that registers RSP-QL queries over streams stored in Solid Pods to produce query results and reuses existing query executions when supported reuse conditions are satisfied. We evaluate Heimdall against Client-Side Processing and a notification intermediary using a wearable-sensor workload with concurrent client scaling, query and data heterogeneity, and concurrent non-reusable queries. For equivalent queries, Heimdall maintains nearly constant latency as client count increases, while Client-Side Processing shows substantial latency growth and instability at higher concurrency. Heimdall also reduces accumulated CPU and memory consumption and client-side network traffic by sharing stream retrieval and query execution. When query execution cannot be reused, shared stream acquisition still improves scalability, although degradation appears as the number of independent queries and physical streams increases with no-reuse. These results show that shared stream acquisition and continuous-query execution can reduce duplicated computation and communication in decentralized stream processing.

## 1. Introduction

A substantial amount of personal data is available digitally. The proliferation of sensor networks, Internet of Things (IoT) devices, and wearable technologies has accelerated the generation of continuous data streams, including physiological and behavioral measurements such as heart rate and location as well as for mobility tracing with vehicle tracking [1,2,3,4]. Extracting timely insights from these streams supports real-time monitoring and decision-making across domains, such as healthcare, smart homes, and smart cities [2,5,6]. A significant portion of this data is stored and controlled by centralized cloud platforms operated by major service providers or corporations, e.g., Meta, Google, or Apple. While centralized infrastructures simplify data collection and analytics, they also create concerns regarding privacy, transparency, data ownership, and interoperability. With changing laws and regulations, e.g., GDPR in Europe [7], privacy is an increasing concern. Since the GDPR became applicable in May 2018, major technology companies, including Google, Amazon, and Meta, have received substantial fines for violations. The largest GDPR administrative fine imposed to date is the €1.2 billion fine issued to Meta Platforms Ireland in 2023 concerning transfers of European Facebook users’ personal data to the United States [8]. The collection of personal data by companies also results in data being duplicated or spread out across multiple applications, e.g., sensor observations from wearable devices, such as heart rate monitors, activity trackers, or smartwatches, are often replicated across multiple cloud services and applications for fitness tracking, healthcare monitoring, and lifestyle analytics. As a result, within a survey in Flanders, 86% of the participants indicated they find it difficult to track which personal information is kept by which company [9]. This leads to fragmented control over personal data. A recent IBM study found that 81% of consumers say that they have become more concerned about how their data is used online. The SolidMonitor indicated that 63% of the participants are worried about their online privacy, with 73% of them being concerned about the lack of transparency of big companies about which data they own about their users [9]. As such, people are also starting to take their online privacy more seriously, with 69% of participants purposely deciding not to share certain data and 66% opting not to use certain applications. This has resulted in a trend where personal data is becoming more and more decentralized as people flock to alternatives to existing services, which provide stronger guarantees regarding privacy, control, and transparency [10].

There is a notable shift towards decentralized storage of personal data in which individuals store their data in general-purpose data stores and can decide at every moment who or which application has access to specific data [11]. This allows users to choose services based on what functionality they provide, rather than where their data is stored. This separation of concerns has the potential to enable trustworthy data storage actors and entirely unburden GDPR compliance for application and service developers. As users have full control over who can access their data, services need to request (temporary) access in case their functionality depends on it.

A key requirement for decentralized data ecosystems is interoperability, realized through universally accepted standards. Technical interoperability ensures that data can be exchanged across systems through common protocols and formats, while semantic interoperability ensures that the meaning of exchanged data remains unambiguous. Semantic Web standards provide a mature interoperable data framework, called the Resource Description Framework (RDF) [12], to link data across organizations using Web technologies, such as global identification via URLs. RDF documents are a collection of triples. Each entry consists of a subject, predicate (i.e., named relation), and an object (or value). As such, RDF allows us to describe how different sensor observations are linked to each other and which properties the data has. Through these linked data principles, data generated by different sensors, devices, and applications is interoperable.

Several decentralized data sharing initiatives have emerged that build upon these interoperability standards and additionally provide mechanisms for identity management, authentication, authorization, and access control. The Solid [13,14] and Linked Web Storage (LWS) [15] initiatives are notable examples. As the most mature initiative, Solid provides personal online data stores, referred to as Pods, in which individuals can manage and control access to their data. For this, Solid uses the Linked Data Platform (LDP) protocol that provides an interoperable folder and documents hierarchy to semantically describe any document using RDF in these Pods, providing the necessary interoperability [16,17]. The Solid vision aims to make data independent of applications through technical specifications, which detail how to publish and consume permissioned data across multiple autonomous personal data storage locations, i.e., Pods.

However, as can be noted from the LDP protocol, the current decentralized initiatives were mainly set up with static data, i.e., documents, in mind. They are ill-prepared to deal with the real-time storage and processing demands of IoT and personal sensor-stream applications. This creates a fundamental challenge: how can decentralized storage solutions support efficient and scalable real-time stream processing without sacrificing user control and privacy. While the remainder of this article will focus on the most mature initiative, i.e., Solid, similar challenges, however, exist across many decentralized storage and data space solutions: while they provide secure and interoperable data management, they are often not optimized for scalable real-time processing of continuously evolving sensor streams.

### 1.1. Problem Statement

  Many decentralized storage solutions, including Solid Pods, primarily focus on secure storage, access control, and interoperability rather than providing built-in stream-processing capabilities. Solid Pods are currently passive data stores and do not execute continuous queries over the data they contain. Consequently, sensor streams stored in Pods must be retrieved and processed by an authorized external client or query-processing service. Although this separation preserves decentralized data ownership and access control, it complicates the continuous computation of up-to-date query results as new stream events arrive [18]. A *view* is a query-defined representation derived from one or more underlying data sources [19]. In real-time monitoring applications, such a view must be continuously updated as the underlying streams evolve. For example, a healthcare provider seeking an overview of a particular health metric across multiple patients must retrieve and process the relevant streams from each patient’s Solid Pod. Because the data is distributed across independently managed Pods, constructing and maintaining such a cross-Pod view introduces communication, coordination, and processing overhead. In a streaming context, view maintenance refers to continuously updating a query-defined result as stream events arrive and expire from the query windows. The maintained result at each evaluation time corresponds to the result obtained by evaluating the continuous query over the current window contents. The problem becomes more pronounced when multiple authorized clients are interested in the same or similar analytics over the same underlying streams. If each client independently subscribes to the Pods, retrieves the stream events, and executes its own continuous query, the same data may be transferred and processed repeatedly. This redundant work increases network traffic, computational resource consumption, query-response latency, and the load placed on the Solid Pods. The central challenge is therefore to efficiently maintain continuously updated query results over streams distributed across multiple Solid Pods while supporting multiple consumers without repeatedly retrieving and processing the same data. In this work, the resulting query updates are delivered directly to subscribed clients and are not persistently stored by the processing service. The decentralized querying process typically results in one of the following three scenarios:

- **One to Many**: One single data Pod updates its data and thus must provide updates to all of the connected consumers, i.e., services. Scalability becomes limited by network bandwidth to send the updates to all the consumers, which also increases the delivery latency.
- **Many to One**: Multiple clients request data from a single data Pod. The data Pod needs to (simultaneously) handle all these requests from all these clients. The single Pod becomes the bottleneck, leading to reduced throughput and increasing response times.
- **Many to Many**: Multiple clients (continuously) request data from multiple data Pods and try to keep the data synchronized and ensure consistency, i.e., keep an up-to-date view. Scalability is restricted due to the additional overhead of synchronization and growth of communication in case each client needs to query all data Pods.

**Figure 1:**
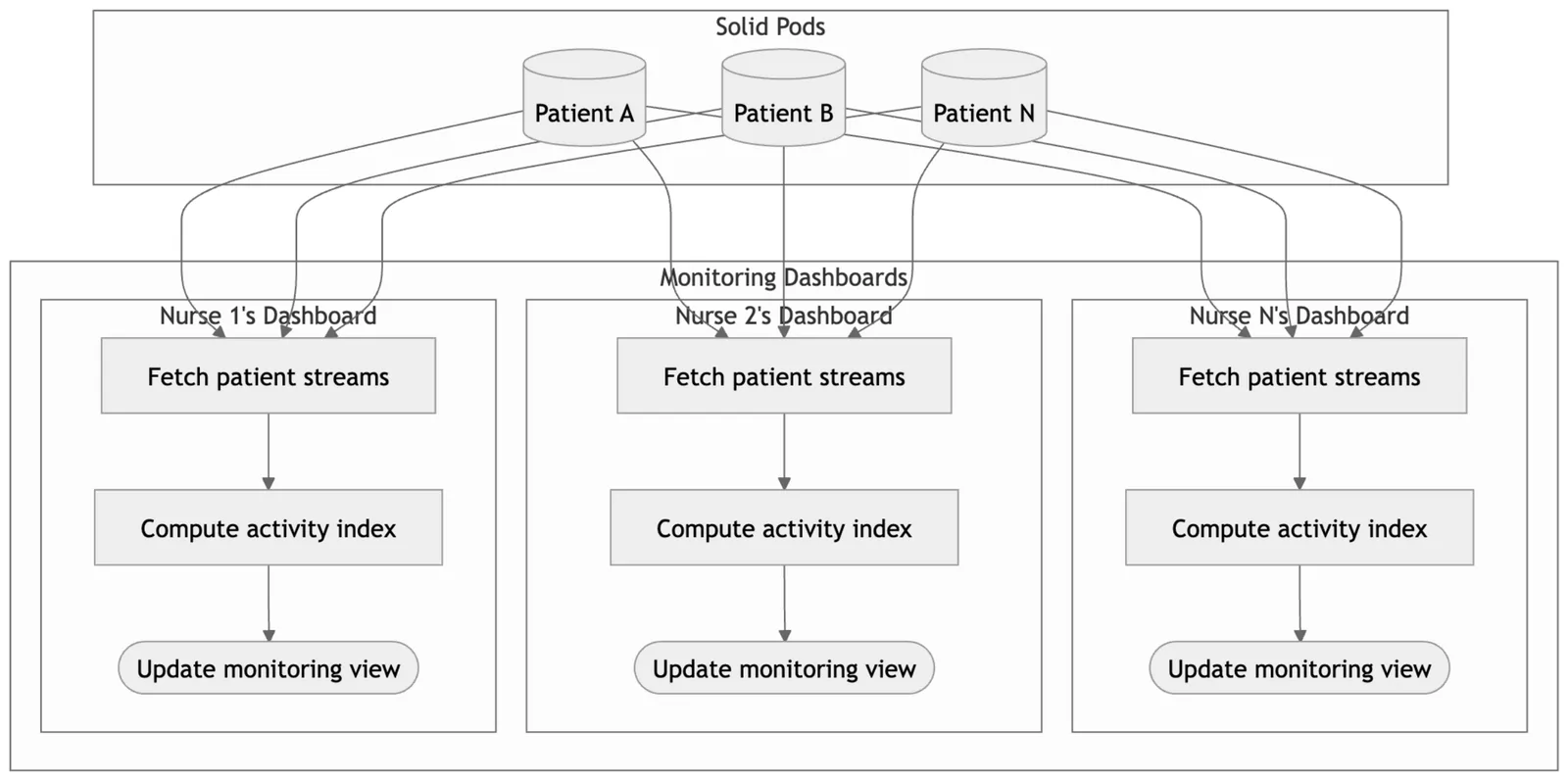
Redundant maintenance of patient-monitoring views without a shared stream-processing service. Each nurse dashboard independently retrieves the same sensor streams from multiple patient Solid Pods, computes the activity index, and updates its own monitoring view by duplicating communication and computation across clients interested in the sensor streams.

Figure 1 illustrates the inefficiencies of maintaining independent query processes for similar continuous queries. Executing equivalent computations separately results in redundant processing, unnecessary network communication, increased resource consumption, and additional load on the decentralized storage systems. These inefficiencies become increasingly significant in sensor-network scenarios where high-frequency data streams require continuous real-time processing. Through our work, we aim to enable scalable and efficient real-time monitoring and management of personal sensor streams stored in decentralized data stores by reducing redundant processing and facilitating the efficient maintenance of continuously updated materialized views.

### 1.2. Research Questions and Contribution

In this subsection, we describe the research questions and the proposed hypotheses guiding our work, as well as the contributions of this paper.

**Research Question 1**: How does the sharing of stream retrieval and continuous-query execution affect latency, resource consumption, and network traffic as the number of concurrent clients requesting queries over Solid-hosted data streams increases?

**Corresponding Hypothesis**: Architectures that share stream retrieval and continuous-query execution will reduce duplicated computation and data transfer compared with architectures in which clients independently retrieve and process data streams, resulting in lower growth in latency, resource consumption, and network traffic as concurrent client load increases.

**Research Question 2**: How do the latency, resource-consumption, and network-traffic trade-offs between Client-Side Processing, partially offloaded processing through a notification intermediary, and shared processing through Heimdall change across (i) the same query over the same data, (ii) different queries over the same data, and (iii) different queries over different data?

**Corresponding Hypothesis**: The benefits of shared processing will depend on which parts of the processing pipeline can be reused. For the same query over the same data, Heimdall can share both stream retrieval and continuous-query execution, reducing duplicated computation and communication. For different queries over the same data, stream retrieval can remain shared while each query requires an independent execution, increasing computational and memory demand while retaining the benefit of shared data acquisition. For different queries over different data, neither query execution nor stream acquisition can be shared, resulting in greater processing and communication demand as the concurrent workload increases.

These research questions are particularly relevant for modern sensor network and IoT deployments where large volumes of distributed sensor data must be collected, processed, and delivered to multiple consumers with minimal latency while maintaining data ownership, privacy, data processing transparency and control.

In this study, we wish to determine if one or multiple clients can efficiently query the personal streaming data stored in the Solid Pod(s) while maintaining low query-response times and efficient resource utilization. This study hypothesizes that, by introducing a Solid Stream Analytics Service, called Heimdall, in the Solid Network, one will be able to efficiently scale to multiple clients in terms of resource usage as well as the query response latency as compared to retrieving and computing the data individually by the clients. The name *Heimdall* refers to the watchman of the gods in Norse mythology guarding Bifrost, the bridge connecting different realms, and continuously watching for approaching events. The name reflects the role of the service as an intermediary between decentralized Solid Pods and the querying clients where Heimdall continuously observes updates to the stream, maintains views, and delivers up-to-date query results to the subscribed clients. This paper introduces Heimdall as a shared stream analytics service which works on top of Solid Pods. However, the idea is also transferable to other decentralized storage solutions. The Heimdall service can be defined as a query agent that continuously maintains materialized views, i.e., up-to-date continuously processed query results, over distributed sensor streams and allows multiple clients to share query results when possible. A query can be quickly evaluated by posting the query to the service rather than computing the query individually by the client. Instead of independently retrieving and processing the same streams, clients can obtain continuously updated results through this service, thereby reducing redundant computation and communication overhead. Moreover, we also wish to determine the optimal strategy in the client-to-analytics service continuum to delegate the stream processing to.

The summarized contributions of this paper are:

- The design and proof-of-concept implementation of Heimdall, an intermediary stream-analytics service that registers and continuously executes RSP-QL queries over streams stored in Solid Pods and distributes the resulting updates to subscribed clients. Heimdall integrates with the Community Solid Server, Solid Notifications, and an RDF Stream-Processing Engine without requiring modifications to the underlying Solid specification.
- A query-reuse mechanism for RSP-QL that identifies reuse-compatible registered queries based on their graph patterns, stream and window semantics, and result semantics, allowing multiple clients to share an existing continuous-query execution and result stream instead of independently retrieving and processing the same input.
- A systematic comparison of three stream-processing architectures across the client-to-cloud continuum—Client-Side Processing, a Notifications Aggregator, and Heimdall—which progressively share stream acquisition and continuous-query execution and thereby allow the effects of processing placement and sharing to be distinguished.
- An experimental evaluation using a realistic wearable-sensor workload that investigates concurrent-client scalability, architectural latency overheads, query and data heterogeneity, and concurrent non-reusable queries. The evaluation measures latency, run completion and processing completeness, CPU and memory consumption, and network traffic, thereby characterizing both the benefits and the limitations of sharing stream acquisition and continuous-query execution.

### 1.3. Paper Organization

The remainder of this paper is structured as follows. Section 2 demonstrates the Heimdall service applied to a realistic use case where personalized data streams need to be processed to showcase the applicability. In Section 3, we introduce the technical background to understand the remainder of this paper. Section 4 discusses the related work. Section 5 describes the architecture, while Section 6 describes the implementation details of the Heimdall service. In Section 7, we describe the evaluation set-up with different evaluation tests. In Section 8, we discuss the evaluation results. Section 9 provides a discussion on the interpretation of the results and the findings. Section 10 finally concludes the main findings of this paper and highlights the future work.

## 2. Running Example

  To illustrate the different components of the architecture and the implementation, we utilize a healthcare-monitoring scenario inspired by the Personalized alaRming and cOntextualized dispaTching through lifEstyle monitorinG (PROTEGO) project [20]. A home-care organization wishes to monitor patients living independently while allowing the patients to retain control over their personal sensor data. In contrast to a traditional centralized approach, raw sensor streams are stored in the patient’s personal Solid Pods rather than copied into a database controlled by the home-care organization. Each patient wears a smartwatch that records movement measurements, such as acceleration across the *x*-, *y*-, and *z*-axes. The measurements are continuously written as events to the patient’s Solid Pod. Authorized healthcare workers monitor these streams to identify situations that may require further investigation, such as prolonged inactivity, unusually erratic movement, or a possible fall. The healthcare workers expect the following functionality from the stream-analytics service:

- The service should enable real-time monitoring of sensor streams stored across one or more Solid Pods.
- Healthcare workers should be able to register and deregister multiple continuous queries over the streams of the patients assigned to them.
- The service should access the private streams through the applicable Solid authentication mechanisms, thereby preserving the patient’s control over access to their data.
- When multiple authorized healthcare workers request the same query, the service should reuse an already executing continuous query instead of independently retrieving and processing the same stream for every worker.

**Figure 2:**
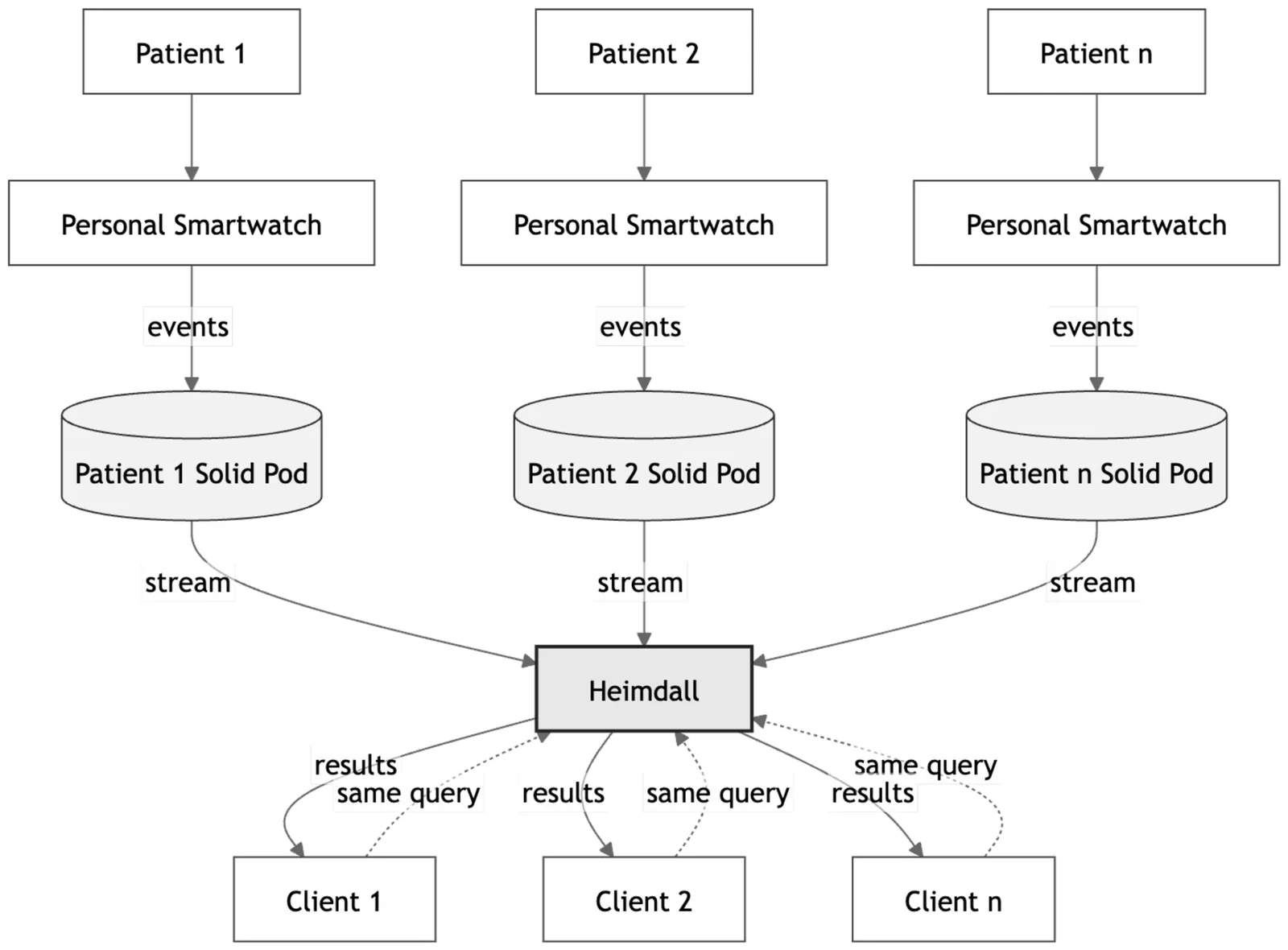
Running example of privacy-preserving patient monitoring with Heimdall. Each patient’s smartwatch writes sensor events to their personal Solid Pod, while multiple clients use Heimdall to execute shared continuous queries over the distributed streams and receive the corresponding results.

In this setting, the same result for a monitoring query can be consumed concurrently by multiple authorized clients. These clients can include healthcare-worker dashboards, mobile applications, friend and family members monitoring the patient, and automated alarming components when there is an anomaly. A single healthcare worker may also use more than one client, for example, a workstation dashboard and a mobile application. Consequently, the number of active clients consuming a monitoring result can be substantially larger than the number of healthcare workers assigned to the individual patient. Each client can register an RSP-QL query for monitoring the patient’s activity. When a newly registered query is reuse-compatible with an already executing query over the same patient streams, Heimdall reuses the existing continuous execution rather than creating another stream-fetching and processing pipeline. The result stream is then dispatched to all clients registered for that query. Figure 2 presents the healthcare-monitoring scenario used throughout this paper. Each patient’s smartwatch continuously generates movement measurements and stores them in the patient’s personal Solid Pod. Multiple authorized monitoring clients can access these distributed streams through Heimdall to receive continuous query results for patient monitoring.

## 3. Background

In this section, we summarize the background knowledge necessary to understand the concepts revolving around the introduced Heimdall service. We first describe Solid as the decentralized storage environment in which the sensor streams are hosted. We then introduce continuous querying, including streams, relations, and window operators, as these concepts provide the foundations for RDF Stream Processing and RSP-QL. Subsequently, we describe RDF Stream Processing and the RSP-QL query model employed by Heimdall. Finally, we introduce out-of-order event processing and watermarks, which are required because events retrieved asynchronously from decentralized Solid resources may arrive out of event-time order. Heimdall is a service that operates on top of the Solid [21] protocol and enables stream processing based on RSP-QL [22]. RSP-QL relies on concepts related to Continuous Querying Language (CQL) [23] and extends the SPARQL [24] query language to enable RDF stream processing. In decentralized Solid deployments, stream events may originate from multiple autonomous Pods and be received asynchronously, causing them to arrive at the stream processor out of event-time order. This motivates the need for out-of-order event handling in our stream-processing approach. It is worth noting that we assume some knowledge on the RDF and SPARQL semantics. We assume that the reader is familiar with the RDF data model and the basic semantics of SPARQL [12,24].

### 3.1. Solid

  Solid [10] is an ecosystem of specifications for interoperable and permissioned access to data stored independently of the applications that consume it. The Solid Project [13] provides the individuals to have more control over the data they generate. Solid [10] aims to utilize the Solid Protocol [21] to facilitate the usage of personalized data vaults called Pods [14]. Data owners can have one or multiple Pods where they can decide the control and who accesses the sensitive personal data streams stored. Solid is built upon existing (Semantic) web standards, as each Pod stores the data in the form of documents containing linked data in the Resource Description Framework (RDF) by exposing HTTP resources organized in containers. Linked data employs the Resource Description Framework (RDF) and the Hypertext Transfer Protocol (HTTP) to publish structured data on the web and connect different data sources, effectively allowing data in one data source to be linked to data in another data source [25]. Solid utilizes both RDF and linked data, as it enables interoperability, extensibility, and reusability among the different actors in the decentralized network. Linked data refers to data published on the web in such a way that it is machine-readable, its meaning is explicitly defined with the typed links, it is linked to other external datasets, and can in turn be linked to from external datasets (such as solid-like data vaults) [26]. RDF is a recommendation of the W3C. The data in RDF follows a triple structure where a subject node is linked to an object node with an edge predicate describing the directed relationship between the nodes. The nodes involved in the graph can either be a named resource (with an Internationalized Resource Identifier (IRI)), an anonymous resource (a blank node) or a literal. Let *I*, *B*, and *L* denote the pairwise disjoint sets of IRIs, blank nodes, and literals, respectively, while *s*, *p*, and *o* denote the subject, predicate, and object of an RDF triple.

**Definition 1.** 
*An RDF triple is a tuple*

(s,p,o)∈(I∪B)×(I)×(I∪B∪L)

Solid becomes an excellent way to store personal data streams, as it supports authentication and authorization out of the box and since it is based upon linked data, it supports interoperability among the service and data providers who are using linked data. In the current form, Solid utilizes LDP as a building block to manage the resources adhering to a REST (Representational State Transfer) manner, making the Solid Pod a huge collection of documents. Solid Resources are retrieved and modified through HTTP methods. One can utilize a SPARQL query to query a particular location in the Pod or use HTTP methods such as GET to query a certain resource or its update. The approach is not optimal for processing streaming data, as the stream keeps evolving with new stream events (in multiple different documents) to process.

#### 3.1.1. Authentication in Solid

Solid follows the Solid OpenID Connect [27] (OIDC) specification to enable authentication. The specification defines how solid servers verify the identity of the users based on the authentication provided by an OpenID provider. Solid-OIDC itself builds on top of OpenID Connect 1.0 [28] and implements it in the Solid ecosystem. In the current fashion, the client registers an account on the Solid Pod with its WebID and an email address with password. The client is redirected to a login screen asking for the email and password. The redirection to a screen to provide consent with email and password is a potential issue for server-only applications with no browser access. The Community Solid Server [29] provides the usage of client credentials [30]. Users can utilize the client credentials to request for a token with which it can authenticate itself without user input. For the headless Heimdall service, we use the Client Credentials mechanism provided by the Community Solid Server. The current Solid-OIDC draft includes the OAuth 2.0 Client Credentials Grant for non-interactive use cases such as scripts, automated agents, and server-to-server communication. In this flow, the client credentials are associated with a WebID, enabling a non-interactive client to obtain the required authentication tokens. However, the credential-provisioning mechanism used by the present Heimdall implementation is provided specifically by the Community Solid Server [29]. Consequently, interoperability currently depends on the Solid server supporting a compatible credential-provisioning mechanism. Supporting and validating interoperable authentication for autonomous agents across different Solid server implementations is therefore considered future work.

#### 3.1.2. Solid Notifications Protocol

The Solid Notifications Protocol [31] provides an extensible HTTP-based framework through which clients can receive notifications about changes to resources. A client first discovers the subscription service available for a resource, requests a notification channel, and subsequently receives messages through that channel. The protocol separates the live-update mechanism from Linked Data Notifications [32], which concern sending and consuming messages through linked data inboxes. The Solid Notifications Protocol is subscription based for a client to receive a notification if the resource on the Solid Pod changes (added, updated, or deleted). The clients can send a subscription request to the Solid Pod to establish a channel to subscribe to for the notifications. Solid Notifications protocol allows different types of notification channels, such as WebSockets, WebHooks, and Streaming HTTP.

### 3.2. Continuous Querying

Continuous querying (CQ) belongs to a specific class of queries which are registered only once and are then continuously executed over the data stream [33]. A continuous query is registered once and evaluated repeatedly as its input data or evaluation conditions change, producing a potentially unbounded sequence of results. Terry et al. [34] introduced the notion of Continuous Semantics by introducing an append-only database, where a query is set to execute repeatedly. Continuous queries process an infinite input, which results in an infinite output. A query’s result is a set of outcomes which would emerge if the query was executed at each instant [35]. Continuous Querying Language (CQL) [23] proposed abstract semantics for continuous queries built upon two fundamental abstractions, i.e., *streams* and *relations* [33]. In this context, a stream *S* represents a potentially unbounded sequence of data items ordered according to a temporal dimension, whereas a relation represents a finite collection of tuples at a particular evaluation time. Continuous-query processing therefore requires mechanisms that derive finite relations from the continuously evolving input stream. CQL decomposes continuous query processing into three classes of operators: Stream-to-Relation (S2R) operators, which derive finite relations from unbounded streams through windows; Relation-to-Relation (R2R) operators, which apply conventional relational operations, and Relation-to-Stream (R2S) operators, which convert changing relations into output streams. The Stream-to-Relation (S2R) operator converts the data stream into data tuples at a specific time through the usage of *Windows*. *Windows* are a set of elements extracted from the stream, denoted as W(S). *Windows* are operators which enable transformation of the unbounded data stream to finite chunks, enabling filtering/analysis and further transformation by the Relation-to-Relation (R2R) operator. CQL defines three different types for Windows. They can either be *time-based*, where the chunks are defined based on time, *tuple-based*, where they are defined based on the amount of RDF objects in the chunk, and *partitioned* window, where the data streams are partitioned based upon the specified *key value* (for example, the ID of the sensor). Within the scope of the current research described in this paper, the focus is on time-based sliding windows. Tommasini et al. [33] define a time-based sliding window as,

**Definition 2.** 
*A time-based sliding window on a stream S takes the time-interval I as a parameter to output a relation R of S [Range I] defined as:*

$$R\left(\tau \right)=\{s|\langle s,\tau^{\prime }\rangle \wedge \tau^{\prime }\leq \tau \wedge \tau^{\prime }\geq \max\left\{\tau -I,0\right\}\}$$

### 3.3. RDF Stream Processing

RDF Stream Processing specializes the continuous-stream model introduced above to streams whose data items are represented as RDF and associated with temporal information [36]. There are different notions of time related with the timestamp. The notions with the most applicability are the time when a data item (such as an RDF object) reaches the data processing system (called the *processing time*), and the time when the data item was produced (called the *event time*).

**Definition 3.** 
*A data stream **S** is an infinite sequence of tuples ($d_{i},t_{e},t_{p}$), where $d_{i}$ is the data item, and $t_{e}/t_{p}$ are the event timestamp and processing timestamp of the event. An **RDF Stream** is a stream where the data item $d_{i}$ is an RDF object and $t_{e}/t_{p}$ are the event timestamp and the processing timestamp of the RDF object, respectively.*

The difference and benefiting factor of RDF Stream Processing is the support for interoperability among multiple heterogeneous data streams by exploiting existing Semantic Web standards.

**Definition 4.** 
*A time-based window operator W is a triple (α,β,$t^{0}$) that defines a series of windows of width (α) and with slide of (β) starting at time $t^{0}$.*

A **Time-Varying** Graph is the result of applying the window operator *W* to an RDF Stream *S*.

**Definition 5.** 
*An **RSPQL dataset** SDS extends the SPARQL dataset [37] with an optional default graph $A_{0}$, n (n ≥ 0) named Time-Varying Graphs, and m (m ≥ 0) named sliding windows over k (k ≤ m) data streams.*

RSP-QL [22] provides a unifying semantic model for expressing and evaluating continuous queries over RDF streams. An RSP-QL query is continuously evaluated against an RSPQL dataset (*SDS*) by an RDF Stream-Processing (RSP) Engine.

**Definition 6.** 
*An RSP-QL Query Q is defined as (SE, SDS, ET, QF), where,*

- *SE is an RSP-QL algebraic expression.*
- *SDS is an RSP-QL dataset.*
- *ET is a sequence of time instants when a query is evaluated.*
- *QF is the Query Form (a SELECT or CONSTRUCT).*

Many RDF stream-processing query engines have been proposed over the years, such as C-SPARQL [38], CQELS [39], and $SPARQL_{stream}$ [40]. RSP4J [41] is a stream-processing system which introduces a flexible API which adheres to the RSP-QL semantics. RSP4J offers the necessary abstractions required for the fast-prototyping of the RSP engines under the RSPQL semantics.

### 3.4. Out of Order Processing

Stream-processing systems commonly distinguish between *event time*, which denotes when an event occurred at its source, and *processing time*, which denotes when the event is processed by the stream-processing system [42,43]. Network delays, asynchronous delivery, retries, and differences in source and processing speeds can cause events to be observed in an order which differs from their event-time order. The original RSP-QL model defines an RDF stream as a potentially unbounded sequence of timestamped RDF statements arranged in non-decreasing timestamp order [22]. Although this assumption simplifies the formal semantics, it does not directly represent deployments in which RDF events can arrive late or out of order. In Heimdall, the situation can occur because notifications and the corresponding RDF resources are received asynchronously from multiple Solid resources or Pods. A common mechanism for reasoning about event-time progress in out-of-order streams is the *watermark* [42,43,44]. A watermark at time τ indicates that the processor considers the event time to have progressed to τ, according to its configured watermark-generation policy. Once the watermark passes the end of a window, the processor may evaluate that dataflow. Events with timestamps behind the watermark are classified as late or may be incorporated, used to revise an earlier result, or discarded depending upon the configured late-event policy or maximum out-of-orderness bound. Systems such as Apache Flink support watermark strategies for streams with bounded out-of-orderness [45]. Heimdall applies a corresponding watermark and maximum out-of-orderness bound policy in its RDF stream-processing engine, as described in the Section 6.

## 4. Related Work

  The related work in this direction of research spans three areas: (1) processing of personal healthcare streams in distributed and decentralized fashion, (2) RDF stream processing, and (3) query processing over decentralized personal data stores. The following subsections describe the existing work conducted in these directions.

### 4.1. Distributed and Decentralized Processing of Healthcare Streams

Distributed and decentralized stream-processing architectures have previously been proposed for processing personal healthcare streams. De Brouwer et al. introduce a distributed Semantic Web architecture for personalized home-care monitoring where relevant sensor streams can be processed close to their sources and personalized patient knowledge can be exchanged across the components [46]. Their subsequent work employs a distributed cascading architecture to derive and manage contextually relevant RDF stream-processing queries for monitoring [47]. Lomotey et al. focus on the traceability of wearable data streams with distributed health information systems [48]. Calbimonte et al. present SemPryv, a decentralized system for semantically enriched personal health streams that allows users to host their data at different sites while controlling access rights [1]. These studies demonstrate distributed or decentralized processing, semantic integration, and personal control over sensitive healthcare streams. However, the present work differs in two different aspects: (1) this work focuses on an interoperable standard such as Solid as the data storage solution and demonstrates continuous querying over data streams with an implementation; (2) this work addresses a different scalability concern, being the duplicated retrieval and processing that occurs when multiple clients independently maintain equivalent continuous query results over data streams stored in decentralized storage solutions such as the Solid Pods. We investigate whether the implementation of an intermediary service such as Heimdall can maintain and share such results while reducing the query latency and resource consumption.

### 4.2. RDF Stream Processing and Stream Publication

RDF Stream Processing (RSP) combines continuous querying over streams with the semantic interoperability provided by RDF. RSP systems such as C-SPARQL, CQELS, and RSP4J provide languages and execution abstractions and engines to enable continuous querying over RDF streams [38,41,49]. Recent surveys classify a broader range of centralized and non-centralized RSP languages, systems, and benchmarks while identifying interoperability, distribution, and scalability as continuing research challenges [50]. Decentralized messaging and stream reasoning-based agent architectures have demonstrated that RSP Engines can exchange RDF streams to coordinate the tasks related to processing the streams [51,52]. Complementary work focuses on publishing and retrieving evolving RDF data. Linked Data Event Streams (LDESs) represent an evolving dataset as an append-only collection of immutable RDF members [53]. Slabbinck et al. demonstrate that LDES event sources can be stored in Solid LDP containers while identifying limitations related to client-managed structure and retention as well as the server-side reorganization with metadata [54]. LDESTS subsequently investigates the compact storage and querying of large time-series streams across Solid Pods [55]. These contributions provide interoperable representation, publication, and storage for RDF streams which evolve over time. They do not, however, provide a query service maintaining a view over access-controlled sensitive streams and distribute results to multiple clients. However, it is possible to reuse the stream publication research conducted in the direction of LDESTS [55] and LDES in LDP [54] to store the streams for historical querying of already materialized queries over the data.

### 4.3. Query Processing over Decentralized Solid Pods

Recent research has investigated query execution over decentralized data across Solid Pods. Link Traversal Query Processing exploits structural assumptions about Solid to discover relevant RDF documents and query without requiring a centralized SPARQL endpoint [56]. Subsequent work has examined practical query interfaces, pruning the sources and describing broader requirements for decentralized query engines including authentication, data discovery, location and schema heterogeneity, and schema alignment [57,58]. Other approaches address specific aspects of decentralized querying. POD-Query employs RDF-to-RDF mapping and query rewriting to expose the schema-aligned views over heterogenous Solid data [59]. Derived resources enable specifying policies over a certain view (with or without a SPARQL query) defined on the server side over the underlying resources in the Solid Pod [60], while ESPRESSO investigates scalable access-aware keyword searches across decentralized Solid Pods [61]. These systems primarily target a single one-shot querying schema alignment rather continuous querying for analytics. Vandenbrande proposes aggregators as query and reasoning agents which maintain up-to-date materialized views over decentralized RDF sources [62] and provides the closest conceptual basis for the current work. Our work specialized this direction for RDF streams and continuous querying over the personal streams stored in Solid Pods. Existing work separately addresses decentralized stream processing, interoperable RDF stream publication, and querying across Solid Pods. However, it does not evaluate how a shared service can continuously maintain and reuse materialized views over private streams in Solid while avoiding duplicate retrieval and computation as the number of authorized clients increases.

## 5. Heimdall

**Figure 3:**
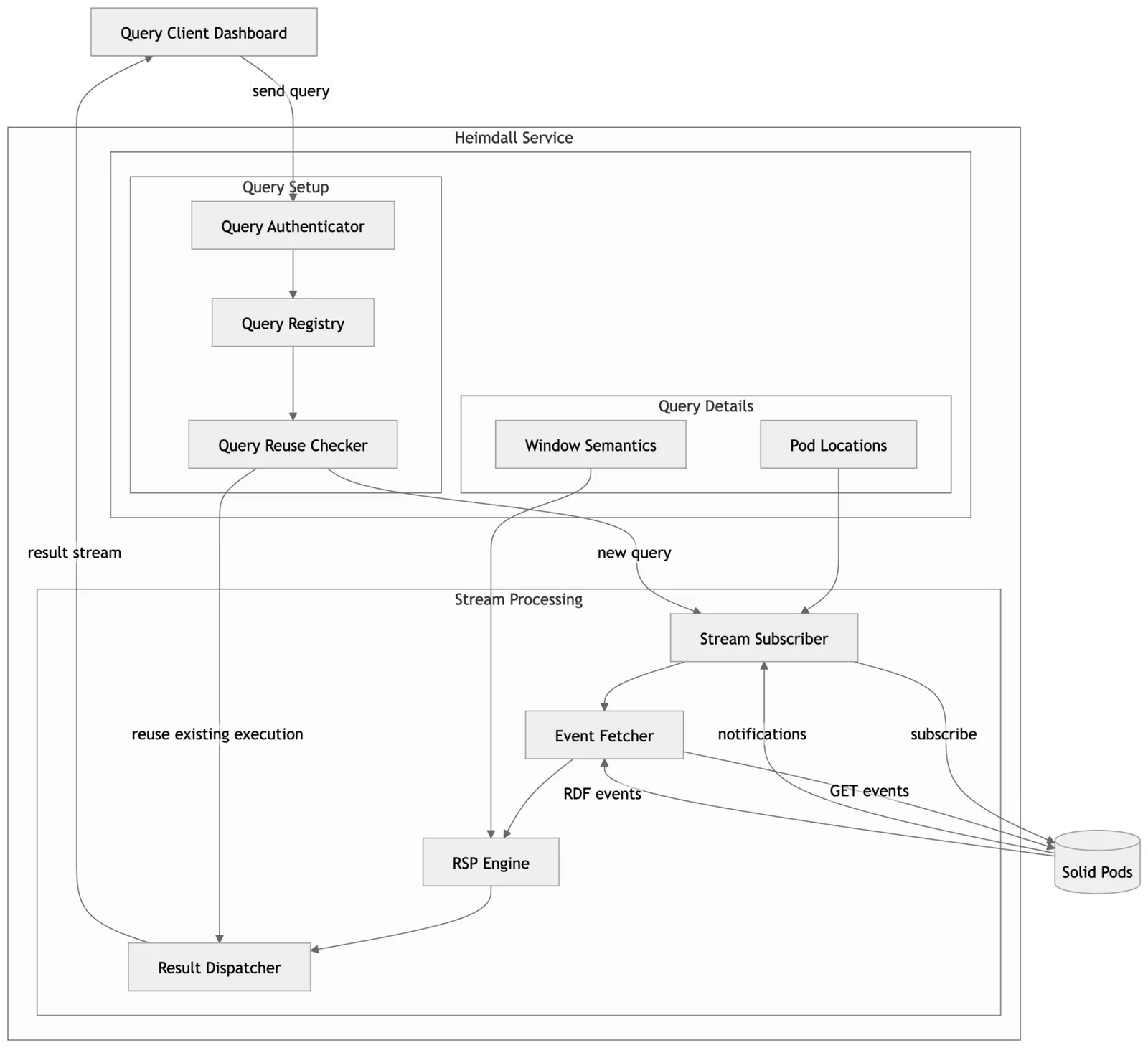
Architecture of the Heimdall service. Query client dashboard registers the continuous queries, which are authenticated, stored, and checked for reuse. Heimdall subscribes to the relevant Solid Pod streams and processes the incoming events using an RSP engine and dispatches the continuous query results to the subscribed clients for the query.

In this section, we describe the architecture of the proof-of-concept Heimdall service (https://github.com/SolidLabResearch/heimdall, accessed on 9 September 2026). As described in Figure 3, Heimdall consists of different components which interact and work together to enable scalable processing of the data streams stored across the Solid Pods. Heimdall is envisioned as an individual stand-alone external service running on top of one or multiple Solid Pods. An external query agent can register a continuous query to maintain a continuous view of the stream stored in the Solid Pod(s). Following the running example we previously introduced here in Section 2, the nurse sends a query to the Heimdall service to monitor the streaming data stored in the patient(s) Solid Pod. Figure 3 decomposes some of the Heimdall components into their main logical responsibilities for clarity. In particular, the Query Reuse Checker and Query Details are functions associated with the Query Registry, while the Stream Subscriber and Event Fetcher are functions of the Notification Stream Processor described in Section 6. An external Query Client Dashboard interacts with Heimdall through a WebSocket interface to register RSP-QL queries and receive continuous query results. The individual components of Heimdall are discussed below:

- *Query Authenticator*: The Query authenticator checks if the query client is allowed to read the sensitive data stream which is stored in the Solid Pod(s). The nurse which registers the query is authenticated with the component to verify if the nurse has the right to read the data from the Solid Pod of the patient(s).
- *Query Registry*: The query sent to Heimdall by the nurse is registered and compared with already executing queries to determine whether an existing query execution can be reused. As illustrated in Figure 3, this involves a Query Reuse Checker, which performs the reuse-compatibility check, and the Query Details required for query execution, including the referenced Pod locations and window semantics. When a reusable query execution is found, its result stream can be shared with the newly registered client, avoiding redundant stream retrieval and query processing.
- *Notification Stream Processor*: The Notification Stream Processor handles the acquisition of stream events referenced by the registered RSP-QL query. Figure 3 shows this component as two logical functions: the Stream Subscriber and the Event Fetcher. The Stream Subscriber subscribes to the relevant Solid resources through the Solid Notifications Protocol. When a notification is received, the Event Fetcher retrieves the corresponding stream event from the Solid Pod using an HTTP GET request, extracts its timestamp, and forwards the timestamped RDF event to the RSP Engine.
- *RSP Engine*: The RSP Engine processes the timestamped RDF stream events according to the registered RSP-QL query and continuously produces the corresponding query results.
- *Result Dispatcher*: The Result Dispatcher maintains the association between executing queries and their subscribed clients. When the RSP Engine produces a result, the dispatcher forwards it to the clients subscribed to the corresponding query execution.

## 6. Implementation of Heimdall

  This section describes the design and architecture choices made for the Heimdall service and details the major components.

### 6.1. Query Authenticator

  Heimdall operates as a separate service layer over the Solid Pod(s). The streaming data stored in the Pod is privacy-centric. From our running example, the patient’s streaming data is private, and not everyone should have access to the patient’s data apart from the trusted entities by the patient. The data owner should have the right to trust a particular service and grant access to the personal data streams and/or other data. After the patient provides access to the Heimdall service, the service can subscribe and request the data stream to develop a materialized view. The Query Authentication component generates an authenticated session for the consumption of the streaming data. In the other implementations of the Solid Server, Solid Pods employ the Solid OIDC spec to establish identity to authenticate and grant access to the data stored in the Solid Pod. However, such flow involves opening up a browser tab for the data owner to give access each time the query is registered. As Heimdall is positioned as a headless back-end service, the current browser-based implementation is not useful in our case. Although the current Solid-OIDC draft defines the Client Credentials Grant for non-interactive actors, the credential-provisioning mechanism used by Heimdall is currently specific to the Community Solid Server. Consequently, portability to other Solid server implementations depends on support for a compatible provisioning mechanism. Supporting and validating interoperable authentication for autonomous agents across different Solid server implementations remains future work. Consequently, the current implementation depends on the Community Solid Server’s [29] credential-provisioning mechanism.

### 6.2. Query Registry

  The Query Registry component of the Heimdall service keeps a log of the multiple queries being registered. The component aims to use the log to find possible overlaps in between the queries to share resources for scalability of the system. In the running example, the nurse(s) and other healthcare workers can register one or multiple queries to the Heimdall service. There are possibilities that the multiple queries registered by the healthcare workers share similar query results and/or are on the same data. The Query Registry component of the Heimdall registers the queries from the different healthcare workers and utilizes them to find similarities between the different query processes such that the aggregated results from one process can be utilized for another healthcare worker if they share the same query. The query registry helps the Heimdall avoid computing the query again. The server can only efficiently handle a certain amount of HTTP requests before being congested. Increasing clients requesting the data on the Solid Server increases the load and decreases the general performance and increase the latency. It avoids the unnecessary extra stream-fetch operations to the Solid Pod, which avoids the congestion of the Solid Pod. The query registry stores the continuous queries in the RSP-QL format [22]. It assigns the registered queries with a query state, such as *registered*, *executing*, and *executed*. All of the queries sent by the healthcare workers are initially assigned with a *registered* state. On the basis of the query registration strategy after the query reuse check, the queries are assigned an *executing* state. After the client de-registers the query, the query is then assigned an *executed* state.

#### Determining Reuse Compatibility Between RSP-QL Queries

Figure 3 represents the query-reuse check and the associated query details as logical functions of the Query Registry. The Query Registry determines whether the execution of an already registered RSP-QL query can be reused for a newly submitted query. We refer to this decision as *query reuse compatibility*. Rather than starting a separate stream processing pipeline for every registered query, the registry compares the new query with the queries that are already being executed. When the comparison succeeds, the new client subscribes to the result stream of the existing query, thereby avoiding duplicate stream retrieval and query execution. The reuse checker performs a structural comparison of the RSP-QL queries. The structural graph-pattern comparison is based on the RDF graph-matching principles described by Carroll [63]. It compares their graph patterns while accounting for consistent variable renaming in the graph pattern and projected expressions. For example, consider two clients monitoring *x*-axis acceleration stream from the running example. The following two RSP-QL queries in Listing 1 use different variable names but are structurally equivalent under a consistent variable mapping:

**Listing 1.** Two reuse-compatible RSP-QL queries from the running example that differ only by consistent variable renaming. % Query A PREFIX saref: <https://saref.etsi.org/core/> PREFIX dahccsensors:  <https://dahcc.idlab.ugent.be/Homelab/SensorsAndActuators/> PREFIX : <https://rsp.js> REGISTER RStream <output> AS SELECT (?accX AS ?value) FROM NAMED WINDOW :w1 ON STREAM :acc_x [RANGE 60000 STEP 20000] WHERE {   WINDOW :w1 {     ?obs saref:hasValue ?accX .     ?obs saref:relatesToProperty       dahccsensors:wearable.acceleration.x .   } } % Query B PREFIX saref: <https://saref.etsi.org/core/> PREFIX dahccsensors:  <https://dahcc.idlab.ugent.be/Homelab/SensorsAndActuators/> PREFIX : <https://rsp.js> REGISTER RStream <output> AS SELECT (?xValue AS ?value) FROM NAMED WINDOW :w1 ON STREAM :acc_x [RANGE 60000 STEP 20000] WHERE {   WINDOW :w1 {     ?measurement saref:hasValue ?xValue .     ?measurement saref:relatesToProperty       dahccsensors:wearable.acceleration.x .   } } 

The two queries differ only in the names of their internal variables. The mapping, μ={?obs↦?measurement,?accX↦?xValue} consistently maps the graph pattern and projected expression of the first query to those of the second query. Both queries reference the same stream and use identical window range and step parameters while exposing the same result variable ?value. The Query Registry therefore considers the queries reuse-compatible and can attach the second client to the result stream of the existing query execution. It also compares the stream and window declarations associated with the graph patterns. In particular, the referenced streams and the window parameters, including the range and step, must match. These conditions are necessary because two queries with structurally similar graph patterns may still process different portions of a stream or be evaluated at different intervals when their window configurations differ. The implemented checker therefore establishes whether the result stream produced by an existing query execution can be shared with a candidate query under the supported comparison criteria. It does not attempt to prove general semantic equivalence between arbitrary RSP-QL queries over every possible input stream. Queries that do not satisfy the reuse conditions are registered as independent query executions.

Algorithm 1 describes the query reuse decision performed by the Query Registry. The algorithm first parses the existing and candidate RSP-QL queries and verifies that both belong to the query fragment supported by the current implementation. It then compares the stream identifier and the temporal window parameters. A difference in the input stream, range, or step causes the queries to process different stream elements or to produce results at different evaluation instants and therefore prevents reuse. The basic graph patterns are subsequently compared modulo consistent variable renaming and triple ordering. The resulting variable mapping is also applied when comparing the result expressions of the queries. Reuse is permitted only when the graph patterns, window semantics, and result semantics match. When the check succeeds, the candidate client is attached to the result stream of the existing execution. Otherwise, the Query Registry creates a separate query execution.
**Algorithm 1** RSP-QL Query Reuse Compatibility Check**Require:** 
Existing executing query $Q_{e}$ and candidate query $Q_{c}$**Ensure:** 
Returns true only when the result stream of $Q_{e}$ can be reused for $Q_{c}$  1:**procedure** CanReuseQuery($Q_{e},Q_{c}$)  2:    $P_{e}\leftarrow$ ParseRSPQL $\left(Q_{e}\right)$  3:    $P_{c}\leftarrow$ ParseRSPQL $\left(Q_{c}\right)$  4:    **if not** SupportedQueryShape($P_{e}$) **or not** SupportedQueryShape($P_{c}$) **then**  5:          **return** false  6:    **end if**  7:    $W_{e}\leftarrow$ GetWindowDefinition(*P*_*e*_)  8:    $W_{c}\leftarrow$ GetWindowDefinition(*P*_*c*_)  9:    **if** $W_{e}.\mathrm{stream}\neq W_{c}.\mathrm{stream}$ **then**10:          **return** false11:    **end if**12:    **if** $W_{e}.\mathrm{range}\neq W_{c}.\mathrm{range}$ **or** $W_{e}.\mathrm{step}\neq W_{c}.\mathrm{step}$ **then**13:          **return** false14:    **end if**15:    **if** $W_{e}.\mathrm{name}\neq W_{c}.\mathrm{name}$ **then**16:          **return** false17:    **end if**18:    $B_{e}\leftarrow$ ExtractBasicGraphPattern(*P*_*e*_)19:    $B_{c}\leftarrow$ ExtractBasicGraphPattern(*P*_*c*_)20:    (matches,μ)← MatchGraphPatterns(*B*_*e*_, *B*_*c*_)21:    **if not** matches **then**22:          **return** false23:    **end if**24:    **if not** SameResultSemantics($P_{e},P_{c},\mu$) **then**25:          **return** false26:    **end if**27:    **return** true28:**end procedure**

### 6.3. Notification Stream Processor

  In Figure 3, the responsibilities of the Notification Stream Processor are represented by the Stream Subscriber and Event Fetcher logical functions. The stream events are written to an LDP container of the Solid Pod with the HTTP protocol with a POST request. The Notification Stream Processor utilizes the Solid Notifications Protocol [31] to subscribe to the latest events written in the *inbox* of the LDP container. The inbox is assigned to the LDP Container and created when the LDP Container itself is created. The subscription request sent by the Notification Stream Processor is an HTTP POST request, which contains the information about the LDP resource to subscribe to (which is the inbox container in our case). The Notification Stream Processor component receives the POST request notification from the Solid Pod regarding the new event which has been added. In the Solid Notifications specification, the new event added is an *Add* event, which differs from the *Update* event when a particular resource is updated with new information or a *Remove* when an event is deleted. At the current moment, we only support the *Add* event, as the stream is an infinite append-only data structure. The support for historical data processing with other events is projected to be implemented in the future. An abbreviated notification received from the Solid Pod is shown in Listing 2. The processor extracts the object field, which identifies the newly added stream resource.

**Listing 2.** Abbreviated Solid notification for a newly added stream event. {   "type": "Add",   "object": "http://localhost:3000/pod1/acc-x/latest_event",   "published": "2023-02-09T15:08:12.345Z" } 

The Notification Stream Processor extracts the *“object”* field and does a GET request to fetch the latest event added to the particular container of the Solid Pod. The container contains the metadata regarding the timestamp path which is used to describe the timestamp of the event. The Notification Stream Processor utilizes the metadata to extract the timestamp of the event with the specified timestamp path (*saref:hasTimestamp* in our case). The event stored in the container is parsed and converted to a Notation3 quad and paired with the extracted timestamp (which is the *event time*). The annotated quad with timestamp is further pushed to the RDF Stream-Processing Engine for stream processing.

### 6.4. RDF Stream-Processing Engine

  The RDF Stream-Processing (RSP) Engine component processes the timestamp annotated quads based on an RSP-QL query to produce the aggregation results. The Heimdall service is RSP Engine agnostic, and different RSP engines can be configured to process the streams from the Solid Pod. In our implementation, we employ the RSP-JS stream-processing engine [64]. We chose the RSP-JS, as it is written in typescript and allows easier and fast prototyping for implementation. The RSP Engine accepts queries in the RSP-QL query format and supports multiple windows and joins over the corresponding stream and static data. The streams referenced by a query may originate from different Solid Pods. Heimdall retrieves the authorized events from the respective stream locations and provides them to the RSP Engine, allowing a continuous query to join data originating from multiple Pods. Input streams are identified by their respective Solid stream locations and may correspond to different LDP containers, including containers hosted in different Solid Pods. Consequently, a single RSP-QL query can define windows over and join stream data originating from multiple Pods. The requests from the different Solid Pods are asynchronous, which makes it difficult to maintain an order or expect ordering in the stream events being ingested to the RSP Engine. Therefore, the Heimdall service needs to utilize an RSP Engine which enables and supports out-of-order handling. The RSP-JS engine supports out-of-order handling with the utilization of watermarks.

#### Out-of-Order Processing

Due to the decentralized nature of the Solid ecosystem, it is entirely possible that there are events which arrive out of order. The RSP Engine for the Heimdall service enables the handling of these out-of-order events utilizing a *watermark*. A watermark represents the temporal completeness of an out-of-order data stream [43]. The Stream-to-Relation (S2R) operator of the RSP Engine processes the existing stream to relations on a timestamp. The out-of-order processing and handling of the events which are late happens in the S2R operator of the RSP Engine. The S2R operator simultaneously maintains two different clocks signifying the flow of the data being ingested in the RSP Engine. The first clock is the *event time* clock, which is based on the events arriving, and the *watermark clock*, denoting the oldest processed timestamp (of the event) for the RSP engine. The watermark therefore denotes that all of the events have been processed, and events before the watermark time should not be considered for processing. The S2R operator of the engine also enables the configuration for a maximum out-of-orderness bound, which is the timestamp range the S2R operator should consider to be added into the window. If a late event arrives to the S2R operator, it is first calculated if the event is in the allowable lateness range. If yes, then it is added to the specific window and the watermark is updated if the event’s timestamp is greater than the current watermark of the engine. The triggering logic of the window is dictated by the watermark clock. At the time when the watermark clock passes the maximum bound of the window, the window is triggered and the window is evaluated by the Relation-to-Relation (R2R) operator to transform the RDF quads of the window based on the RSP-QL query sent by the nurse (the client).

Heimdall applies a bounded out-of-order event-processing policy with single-emission windows. Let *M* denote the maximum event timestamp observed by the S2R operator and let *B* denote the configured maximum out-of-orderness bound. The watermark is calculated as ω=M−B. A window remains active until the watermark reaches its closing timestamp. During this period, an event may be added to the window even when it arrives after events with later timestamps, provided that its event timestamp belongs to the window and it is not behind the watermark. Once the watermark reaches or passes the closing timestamp of the window, the window is evaluated once, its result is emitted, and the state is removed from the memory and not maintained. The implementation does not retain the finalized window state and does not revise or re-emit the previously produced results. Consequently, an event belonging to an already finalized window is therefore considered too late and is thus discarded. The configured bound therefore determines how long the system waits for out-of-order events before finalizing a window. Therefore, the current system does not wait to cause additional result latency and makes a trade-off with the possible missing delayed event which was supposed to arrive but did not arrive on time. Algorithm 2 describes this process on how the S2R operator processes each incoming event according to its event timestamp. For each incoming event *e* with event timestamp $t_{e}$, the operator updates the maximum observed event time and derives the current watermark. The events behind the watermark are discarded. Otherwise, the event is then inserted into each active window which is applicable for the event based on the timestamp. Windows whose closing timestamps have been reached by the watermark are subsequently evaluated, emitted with the R2R operator, and removed from the active-window state. To give an example, an event with timestamp $t_{e}=1$ can still be included when it arrives after events with timestamps up to 29, considering the maximum out-of-order bound is 30, as long as the watermark has not finalized the event’s corresponding window. If the same event arrives after the window has been finalized, it is discarded because the window state is no longer available. An update-aware extension could retain a finalized window for an additional configurable correction interval rather than immediately removing its state. During this interval, an event whose event timestamp belongs to the finalized window could be inserted into the retained window state and the corresponding R2R operation could be re-evaluated. If the resulting query result differs from the previously emitted result, the Result Dispatcher could emit either a retraction followed by the corrected result or a versioned replacement result. After the correction interval expires, the retained window state would be discarded permanently. Such a policy would increase tolerance for very late events at the cost of additional memory consumption, repeated query evaluation, and a longer period before a result can be considered final.
**Algorithm 2** Bounded Out-of-Order Event Handling with Single-Emission Windows**Require:** 
Event *e* with event timestamp $t_{e}$**Require:** 
Maximum out-of-orderness bound *B***Require:** 
Maximum observed event time *M*, active windows A**Ensure:** 
The event is inserted into applicable non-finalized windows or discarded  1:**procedure** ProcessEvent($e,t_{e}$)  2:      $M\leftarrow \max(M,t_{e})$  3:      ω←M−B  4:      **if** $t_{e}<\omega$ **then**  5:            DiscardAsTooLate(*e*)  6:            **return**  7:      **end if**  8:      CreateRequiredActiveWindows($t_{e},\omega ,A$)  9:      **for all** w∈A **do**10:            **if** $w.\mathrm{open}\leq t_{e}<w.\mathrm{close}$ **then**11:                 AddEvent(w,e)12:            **end if**13:      **end for**14:      **for all** w∈A ordered by w.close **do**15:            **if** w.close≤ω **then**16:                 $R_{w}\leftarrow$ EvaluateWindow(*w*)17:                 EmitResult($R_{w}$)18:                 A←A∖{w}19:            **end if**20:      **end for**21:**end procedure**

## 7. Evaluation

  This section evaluates whether Heimdall improves the scalability and efficiency of continuous stream processing over the data stored in Solid Pods. We conduct controlled experiments using real-world wearable-sensor traces that are replayed as live RDF streams to a Community Solid Server. The same continuous query, input stream, RDF Stream-Processing Engine, and the stream-processing logic are used across all evaluated approaches. Consequently, the differences primarily result from where notification handling, event retrieval, and query processing are performed.

This evaluation addresses four questions. First, we examine how increasing the number of concurrent clients affects latency, resource consumption, and network traffic. Second, we quantify the one-time and recurring latency overheads introduced by the different architectures. Third, we investigate whether the architectural trade-offs change when the query and/or underlying data differ. Finally, we examine the behavior of Heimdall under increasing concurrent load when query execution cannot be reused.

### 7.1. Evaluation Questions and Metrics

The experiments for evaluation are organized around the following evaluation questions:

- **EQ1**: How does increasing concurrent client load affect latency, CPU utilization, memory consumption, and the network traffic across the stream-processing architectures?
- **EQ2**: What one-time and recurring latency overheads are introduced by the different stream-processing architectures?
- **EQ3**: How does varying query and data similarity affect the latency and resource consumption trade-offs between the stream-processing architectures?
- **EQ4**: How does Heimdall scale as the number of concurrent independent queries increases under different degrees of stream sharing, and when does processing begin to degrade?

**EQ1** and **EQ2** primarily address Research Question 1. **EQ1** evaluates the effect of increasing concurrent client load, while **EQ2** identifies the architectural operations contributing to the observed latency differences.

**EQ3** addresses Research Question 2 by examining how query and data heterogeneity affect the three stream-processing architectures under controlled single-client conditions.

**EQ4** primarily addresses Research Question 2 by evaluating how Heimdall scales when opportunities for query-execution reuse and stream-acquisition sharing are progressively reduced.

To answer these evaluation questions, we measure first-result latency, CPU utilization, resident memory consumption, processing completeness, and network traffic where applicable. The latency, resource-consumption, and network-traffic measurement procedures are described in the following subsections.

### 7.2. Compared Stream-Processing Approaches

**Figure 4:**
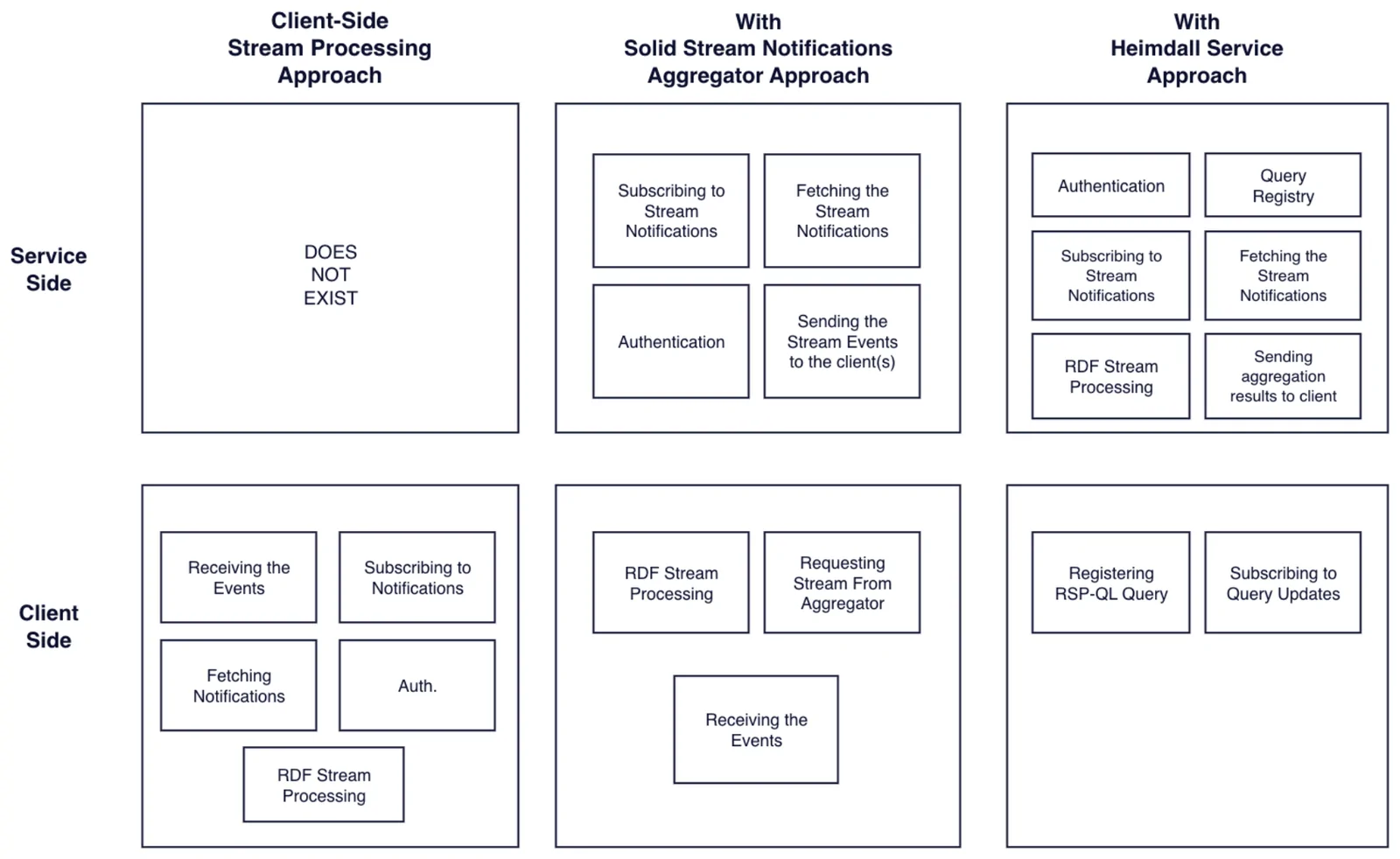
Distribution of stream-handling responsibilities between the service and client sides for the three evaluated approaches: Client-Side Stream-Processing approach, processing with the Solid stream-notifications aggregator, and processing with the Heimdall service.

We compare three approaches that place the stream-processing responsibilities at different positions in the Client-to-Cloud continuum. Figure 4 summarizes the distribution of responsibilities between the service and client sides. Although, the figure shows one client for clarity, and each approach supports multiple clients in the experiments.

- **Client-Side Processing**: Each client independently subscribes to the relevant Solid notifications, retrieves every newly added RDF resource, and executes the continuous query locally with an RSP Engine. This approach does not use an intermediary service.
- **Solid Stream-Notifications Aggregator**: A shared intermediary (https://github.com/SolidLabResearch/solid-notifications-aggregator, accessed on 9 September 2026) subscribes to the Solid Pod and retrieves each new stream event once. It forwards the retrieved events to subscribed clients interested in the same stream through WebSockets. Each client nevertheless executes the continuous query independently.
- **Heimdall**: Heimdall subscribes to the Solid Pod, retrieves the stream events, and executes the continuous query centrally. When multiple clients register an equivalent query, Heimdall reuses the existing query execution and distributes the resulting stream to all subscribed clients.

The three approaches use the same RSP engine and equivalent notification parsing, timestamp extraction, and query evaluation logic, as well as the continuous query being evaluated.

### 7.3. Evaluation Workload

The evaluation instantiates the lifestyle-monitoring scenario introduced in Section 2. Accelerometer observations from a wearable device are stored as RDF stream events in a participant’s Solid Pod. Authenticated clients continuously calculate an activity index from the acceleration measurements along the *x*-, *y*-, and *z*-axes. Following the HEAVEN characterization of RDF Stream-Processing experiments  [65], the evaluated processing systems correspond to the three stream-handling approaches, the input data is the DAHCC accelerometer stream, the workload uses DAHCC, SAREF, and LDP vocabularies, the query calculates the activity index, and the reported KPIs comprise latency, CPU utilization, and memory consumption.

#### 7.3.1. Dataset and RDF Representation

The workload is derived from the Data Analytics for Health and Connected Care (DAHCC) dataset [66,67]. The dataset contains sensor observations collected in imec’s HomeLab environment as part of the PROTEGO project [20,68]. We use the raw wearable data of the anonymized sixth participant because this participant’s trace provided the most complete acceleration measurements among the available data. The selected measurements contain acceleration values along the *x*-, *y*-, and *z*-axes. A mapper script converts the source data into RDF observations using the DAHCC and SAREF ontologies [69,70]. Each observation contains its measurement value, timestamp, originating sensor, and measured property. The complete mapping implementation and generated workload are available in the accompanying repository.

#### 7.3.2. Continuous Query

All approaches execute the same RSP-QL query. The query creates three time-based sliding windows over the acceleration streams and calculates the activity index with the *x*-, *y*-, and *z*-measurements. Each window has a range of 60 s and a step of 20 s. The result is emitted as an RStream from the RSP Engine. We envision the dashboards to either display this result or utilize the result for anomaly detection. The query utilized for the scenario to calculate the activity index is described in Listing 3.

The query computes the activity index with the accelerometer sensor from three-axis streams. The func:sqrt function is not part of the standard SPARQL function set. It is an implementation-specific extension function provided by the employed RSP engine and is identified by the http://extension.org/functions#sqrt IRI. The function accepts a numeric argument and returns its square root. The three evaluated approaches use the same extension function implementation and the RSP engine.

**Listing 3.** RSP-QL query used to compute per-sample activity-index values from synchronized three-axis accelerometer streams. The func:sqrt expression is an implementation-specific extension function provided by the employed RSP engine. PREFIX saref: <https://saref.etsi.org/core/> PREFIX func: <http://extension.org/functions#>
PREFIX dahccsensors:  <https://dahcc.idlab.ugent.be/Homelab/SensorsAndActuators/> PREFIX : <https://rsp.js> REGISTER RStream <output> AS SELECT (   func:sqrt(     ?accX * ?accX +     ?accY * ?accY +     ?accZ * ?accZ   ) AS ?activityIndex ) FROM NAMED WINDOW :w1 ON STREAM :acc_x [RANGE 60000 STEP 20000] FROM NAMED WINDOW :w2 ON STREAM :acc_y [RANGE 60000 STEP 20000] FROM NAMED WINDOW :w3 ON STREAM :acc_z [RANGE 60000 STEP 20000] WHERE {   WINDOW :w1 {     ?s1 saref:hasValue ?accX .     ?s1 saref:hasTimestamp ?timestamp .     ?s1 saref:relatesToProperty dahccsensors:wearable.acceleration.x .   }   WINDOW :w2 {     ?s2 saref:hasValue ?accY .     ?s2 saref:hasTimestamp ?timestamp .     ?s2 saref:relatesToProperty       dahccsensors:wearable.acceleration.y .   }   WINDOW :w3 {     ?s3 saref:hasValue ?accZ .     ?s3 saref:hasTimestamp ?timestamp .     ?s3 saref:relatesToProperty       dahccsensors:wearable.acceleration.z .   } } 

#### 7.3.3. Stream Replay

The mapped observations are stored in an N-Triples input file and replayed to the Solid Pod using the Stream-Replayer Service [71]. The replayer creates the required LDP containers and submits observations using HTTP POST requests. Because the source dataset contains historical timestamps, the replayer assigns new event timestamps relative to the beginning of each experimental run. At a replay frequency of 4 Hz, consecutive observations are submitted every 250 ms. All evaluated approaches receive the same event sequence and replay rate.

The replay workload is deterministic rather than bursty where observations are emitted at fixed intervals according to the configured replay frequency. Consequently, the original inter-arrival times and burstiness of the recorded sensor trace are not preserved. Similarly, while the values of the observations originate from the recorded dataset, their event timestamps are reassigned according to the replay schedule. This controlled replay ensures that all evaluated approaches are exposed to the same reproducible input workload. The evaluation therefore characterizes the considered architectures under a fixed-rate workload, and the effect of bursty or dynamically varying event rates is outside the scope of the present evaluation.

### 7.4. Experimental Platform

This subsection describes the hardware, network, and software environment used to conduct the evaluation. The experimental platform was designed to isolate the main system components and minimize interference between the Solid Pod, the Stream Replayer, the evaluated stream-processing service, and the clients.

#### 7.4.1. Hardware and Network

The experimental deployment uses four physical machines. Each machine is assigned one of four roles: (1) the Solid Server, (2) the shared processing service (Heimdall or the Notification Aggregator), (3) the Stream Replayer, or (4) the Query Clients. Separating these roles prevents competition for CPU and memory resources between the principal architectural components and enables process- and host-level resource measurements.

In the concurrent multi-client experiments, all client processes execute simultaneously on the dedicated client machine, while the Solid Server, shared processing service, and Stream Replayer execute on separate physical machines. Consequently, increasing the number of clients introduces contention between client processes on the shared client host, while resource interference between the principal architectural roles remains isolated. This configuration therefore captures contention-aware multi-client behavior, including CPU scheduling and shared client-host resource pressure.

Because real deployments may instead execute clients on independently provisioned devices, the evaluation additionally reports a distributed-client resource projection that isolates the accumulation of computation and memory from contention caused by co-locating the experimental clients on one physical machine. The projection is therefore complementary to, rather than a replacement for, the direct concurrent measurements.

The machines are connected through a dedicated local-area network. No unrelated application traffic is generated on the network during the experiments. Each machine runs Ubuntu 22.04.5 LTS and is equipped with an Intel(R) Core(TM) i5-9500 CPU @ 3.00 GHz, comprising six physical cores, and 62 GiB of RAM.

**Figure 5:**
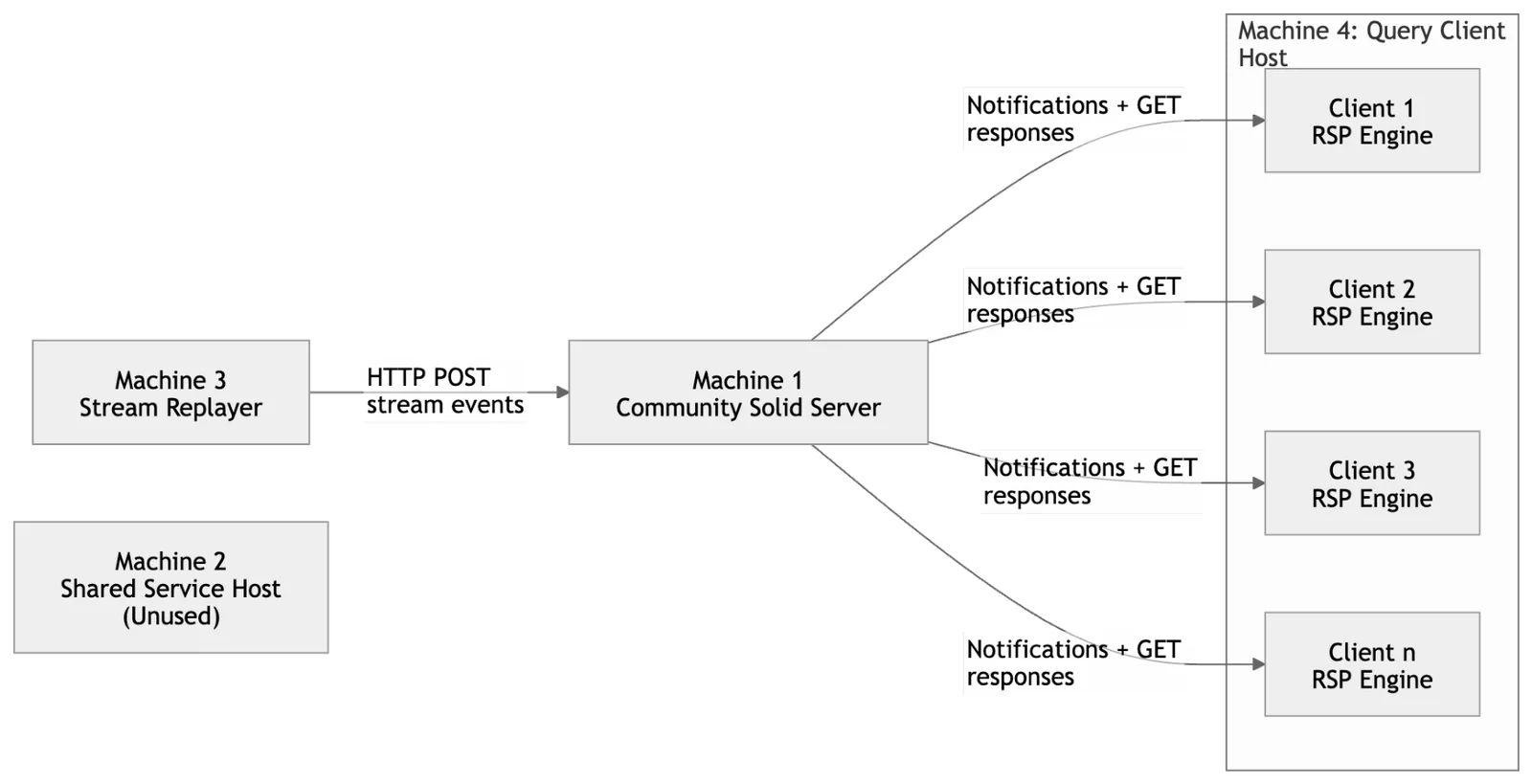
Experimental deployment for Client-Side Processing. All concurrent client processes execute on the dedicated client host. Each client independently subscribes to the Solid streams, retrieves newly published events, and executes an independent RSP query-processing pipeline. The shared-service host is not used in this approach.

**Figure 6:**
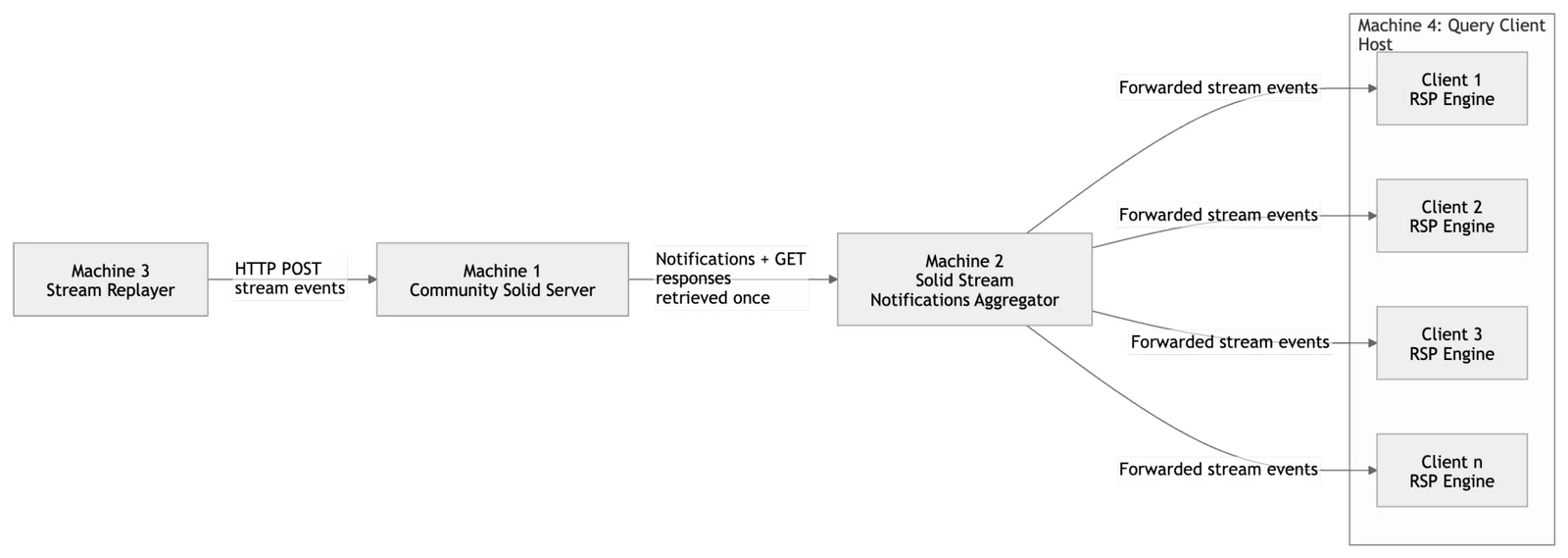
Experimental deployment for the Notification Aggregator. The aggregator subscribes to the Solid streams and retrieves each newly published event once before forwarding the retrieved stream events to all subscribed clients. All concurrent clients execute on the dedicated client host, where each client maintains an independent RSP query-processing pipeline.

**Figure 7:**
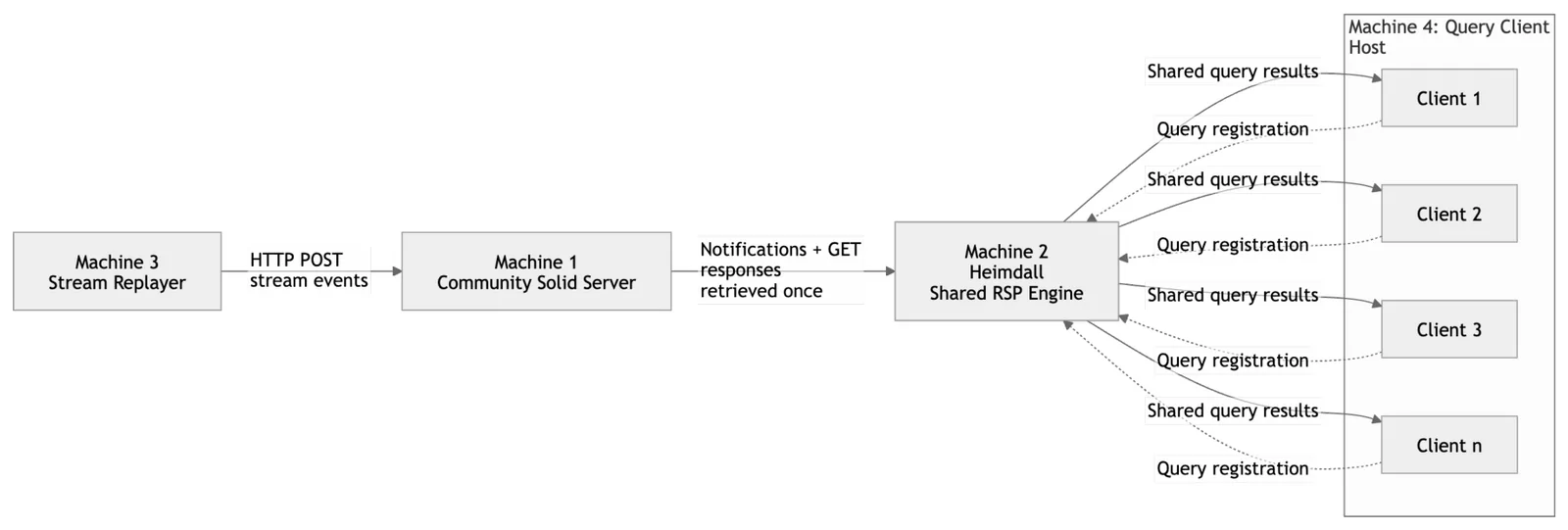
Experimental deployment for Heimdall. Heimdall subscribes to the Solid streams, retrieves each newly published event once, and maintains a shared RSP query execution for reuse-compatible queries. Concurrent clients execute on the dedicated client host and register their queries with Heimdall. When an existing query execution can be reused, Heimdall distributes the shared result stream without forwarding the complete input stream or creating an additional RSP query-processing pipeline.

Figure 5, Figure 6 and Figure 7 show the physical deployment used for the three evaluated processing approaches. The same four-machine allocation is retained across the experiments. The shared-service machine is unused for Client-Side Processing, hosts the Notification Aggregator in the second approach, and hosts Heimdall in the third approach.

#### 7.4.2. Solid Pod Configuration

The evaluation uses the Community Solid Server [29]. It is configured with file-system-backed storage, Redis-based locking, Webhook notification channels, and multiple HTTP workers. The lock-expiration duration is set to 180 s. Three LDP containers, /pod/acc-x/, /pod/acc-y/, and /pod/acc-z/, store the acceleration streams. Their locations are registered in the Pod’s Public Type Index [72]. Service authentication is performed using Community Solid Server’s client credentials. The source code, configuration files, and workload generation scripts are publicly available in the accompanying repository.

### 7.5. Measurement Methodology

Before every experimental repetition, the query-processing services and clients are restarted and their internal state is cleared. The same input stream, query, replay frequency, Solid Pod configuration, and network topology are used across the three evaluated approaches. Each experimental configuration is executed for 35 repetitions. Following established performance-evaluation practices that separate the warm-up behavior from the observation phase [73,74,75], we exclude the first three iterations (*r01–r03*) as warm-up iterations and the final two repetitions (*r34–r35*) as cool-down repetitions.

A retained repetition is additionally classified according to the applicable completion criterion of the experiment. For **E1**, repetitions in which the evaluated architecture fails to complete successfully remain part of the reported run-completion rate but are excluded from latency and other summaries that require a successfully completed execution. In **E4**, by contrast, incomplete physical-input processing is itself an experimental outcome used to characterize scalability. Such retained repetitions therefore remain included in the latency and network summaries rather than being removed because of their incompleteness. Resource measurements are included when the corresponding process trace covers the required measurement interval; where fewer than 30 traces satisfy this criterion, the number of included repetitions is reported explicitly. Unless otherwise indicated, quantitative results are reported as the arithmetic mean and standard deviation across the repetitions applicable to the corresponding metric.

#### 7.5.1. Run Completion Measurement

For every client-count and architecture configuration, the number of successfully completed repetitions is recorded relative to the 30 retained attempts. Run completion is reported independently from latency and resource measurements so that degradation resulting in unsuccessful execution is not hidden by statistics calculated only over successful repetitions. The success rate is calculated as

(1) $$S=\frac{N_{\mathrm{successful}}}{N_{\mathrm{attempted}}}\times 100,$$

where $N_{\mathrm{attempted}}=30$ for every evaluated configuration.

#### 7.5.2. Latency Measurement

The end-to-end latency measurements in E1, E3, and E4 use the interval between the first stream event entering the query-processing pipeline and production of the first continuous-query result. E2 does not use this end-to-end metric to compare scalability. Instead, it decomposes the processing path into the individual one-time and recurring operations described in Section 7.6.2.

The evaluated RSP-QL query uses a window step of 20,000 ms. Consequently, the measured first-event-to-first-result interval contains a fixed 20 s semantic waiting period before the first window evaluation can produce a result. To isolate the additional delay introduced by the processing architecture and concurrent system load, we therefore report the *first-result latency*,

(2) $$L=\left(t_{\mathrm{first}\;\mathrm{result}}-t_{\mathrm{first}\;\mathrm{event}}\right)-20,000\;\mathrm{ms}.$$

This metric does not represent the execution time of the RSP engine alone. Instead, it captures the additional end-to-end delay resulting from notification delivery, event retrieval, scheduling, query processing, and other architectural overheads beyond the fixed window-step interval.

#### 7.5.3. Resource-Usage Measurement

CPU utilization and Resident Set Size (RSS) are sampled throughout each experimental repetition for the concurrently executing client processes and, where applicable, the shared Heimdall or Notification Aggregator process. For each client process, mean CPU utilization and mean RSS are calculated over the measurement interval. CPU utilization is reported using the process-level multi-core convention, where 100% corresponds to the full utilization of one CPU core and, consequently, a multi-threaded process may report utilization greater than 100%. CPU utilization and Resident Set Size (RSS) are sampled at 500 ms intervals throughout each experimental repetition for the concurrently executing client processes and, where applicable, the shared Heimdall or Notification Aggregator process. The directly measured accumulated client-process resource consumption for a repetition is then calculated as

(3) $$R_{\mathrm{clients}}\left(n\right)=\sum_{i=1}^{n}R_{c_{i}},$$

where $R_{c_{i}}$ denotes the measured mean CPU utilization or RSS of client process *i* during that concurrent repetition.

For architectures containing a shared service, its resource consumption $R_{s}$ is measured separately on the dedicated service machine. Client-process and service-process measurements are therefore retained separately in the reported results. Where an accumulated process footprint is reported for descriptive comparison, it is calculated as

(4) $$R_{\mathrm{processes}}\left(n\right)=R_{s}+\sum_{i=1}^{n}R_{c_{i}}.$$

These values are direct measurements of processes executing during the concurrent experiment rather than extrapolations from isolated single-client executions. For CPU utilization, the accumulated process value should be interpreted as process-level computational demand across the participating hosts rather than as utilization of a single physical CPU. Because all concurrent client processes execute on one dedicated client machine, the experiment also records host-level CPU utilization of that machine. This measurement captures the actual CPU pressure experienced by the shared client host as the number of concurrent processes increases.

#### Decentralized-Client Resource Usage Projection

The direct concurrent measurements include contention between client processes because all clients execute on the same physical client host. In a decentralized deployment, individual clients may instead execute on independently provisioned devices. We therefore additionally calculate a distributed-client resource projection to isolate architectural resource duplication from client-host contention.

Let $R_{c}$ denote the resource consumption measured for one independently executing client and $R_{s}$ the corresponding shared-service resource consumption. The projected accumulated resource demand is calculated as

(5) $${\hat{R}}_{\mathrm{client}}\left(n\right)=nR_{c}$$

for Client-Side Processing and

(6) $${\hat{R}}_{\mathrm{shared}}\left(n\right)=R_{s}+nR_{c}$$

for architectures containing a shared service.

The projection is applied independently to CPU utilization and RSS. It represents the accumulated resource footprint when client resources are assumed to be independently provisioned and therefore does not include contention between client processes on a shared machine. The projection is not used as a prediction of concurrent latency or saturation and does not remove possible contention at shared components such as the Solid server, network, or intermediary service.

#### 7.5.4. Network-Traffic Measurement

Network traffic is measured independently at the experiment-facing interfaces of the Solid-server host, shared-service host, client host, and Stream Replayer. For each measurement interval, the transmitted and received byte-counter differences are converted to average bandwidth as

(7) $$B_{\mathrm{TX}}=\frac{(TX_{\mathrm{end}}-TX_{\mathrm{start}})\times 8}{\Delta t\times 10^{6}},$$

(8) $$B_{\mathrm{RX}}=\frac{(RX_{\mathrm{end}}-RX_{\mathrm{start}})\times 8}{\Delta t\times 10^{6}},$$

where $B_{\mathrm{TX}}$ and $B_{\mathrm{RX}}$ are expressed in Mbps and Δt denotes the measurement duration in seconds.

The traffic measured at different hosts is not summed into a network-wide total, because the same packet may cross multiple measured interfaces and would therefore be counted more than once. Instead, the measurements are interpreted according to their architectural boundaries. In particular, Solid-server transmission quantifies upstream load generated at the decentralized storage component, client-host reception quantifies the traffic delivered to the client side, and service–host transmission and reception characterize traffic through the shared intermediary. The per-client network demand is reported by dividing aggregate bandwidth by the number of concurrently active clients.

### 7.6. Experiments

#### 7.6.1. E1: Scalability Under an Increasing Number of Clients

The experiment evaluates EQ1 by increasing the number concurrent active clients and studying its effect on the stream-processing architectures, which are Client-Side Processing, Notifications Aggregator, and Heimdall. The clients request the same activity-index query over the same input streams. In Heimdall, the stream retrieval and query execution are shared. While the Notification Aggregator shares the stream retrieval where each client independently executes an RSP pipeline. With Client-Side processing, both retrieval and the query execution are performed independently by every client. The concurrent experiment measures first-result latency, successful completion, client and service CPU utilization, RSS, and network traffic at the client host, intermediary-service host, and Solid server. It is worth noting that all client processes execute concurrently on the same dedicated client machine. This provides a contention-aware measurement of the three stream-processing architectures. A complementary, more realistic decentralized-client resource projection is subsequently calculated from independently measured client and service resource consumption. This projection represents the accumulated computational and memory requirements when client processes are assumed to execute on independently provisioned hosts and is used to distinguish architectural duplication from contention introduced by co-locating the experimental clients.

#### 7.6.2. E2: Latency Decomposition

This experiment evaluates EQ2 by comparing the latency components of all three approaches for a single client. The measurements distinguish operations executed once during query initialization from operations repeated for each event or window. The one-time measurements include stream discovery, service discovery where applicable, authentication, query reuse checking, query registration, stream subscription, and WebSocket establishment. The recurring measurements include event retrieval, RDF parsing and timestamp extraction, insertion into the RSP engine, window-query evaluation, and result delivery to the clients interested in the results. The experiment also records the proportion of events that arrive out of order and their observed lateness under the configured maximum out-of-orderness bound (which was 30 s in the current experiment configuration). A single client is used deliberately in E2 because the objective of this experiment is to isolate and compare the latency introduced by individual architectural operations rather than to evaluate scalability with client count. The effect of increasing the number of concurrent clients on end-to-end latency is evaluated separately in E1.

#### 7.6.3. E3: Query and Data Heterogeneity

This experiment evaluates EQ3 by isolating the effect of query and data heterogeneity from the effect of concurrent client load. We compare the three stream-processing architectures under three workload scenarios: (i) the same query over the same data, (ii) different queries over the same data, and (iii) different queries over different data.

Three controlled RSP-QL query variants are employed. All variants preserve the same continuous-query structure, consisting of three named input windows with a 60 s range and 20 s step and the same activity-index calculation, while varying the RDF triple pattern used to select the observations. The variants, respectively, select observations according to the measured property, the producing sensor, and the measurement type. For the different-data workload, the corresponding queries additionally operate over different segments of the input stream.

A single client is used for each run to isolate heterogeneity from the contention effects evaluated in **E1** and **E4**. The experiment measures the latency, client and service CPU utilization, RSS memory consumption, and network traffic.

#### 7.6.4. E4: Scalability Under Concurrent Non-Reusable Queries

This experiment evaluates **EQ4** by increasing the number of concurrently executing query instances within Heimdall while preventing query-execution reuse. Unlike **E1**, where multiple clients register reuse-compatible queries and can therefore share an existing continuous-query execution, **E4** deliberately creates query instances that are considered non-reusable by the current Query Registry.

To isolate the scalability cost of maintaining additional independent RSP executions from differences in query complexity, the query logic, graph pattern, referenced streams, window range, window step, and result semantics are kept unchanged across the query instances. The instances differ only in their named-window identifiers. For example, one query may define and reference the window :q1, while another otherwise identical query uses :q2.

For example, in Listing 4, consider two query instances over the same acceleration streams. The relevant window declarations may differ as follows:

**Listing 4.** Example of two non-reusable RSP-QL query instances used in E4a. The instances differ only in their named-window identifiers. % Query instance Q1 FROM NAMED WINDOW :q1_wx ON STREAM :acc_x [RANGE 60000 STEP 20000] FROM NAMED WINDOW :q1_wy ON STREAM :acc_y [RANGE 60000 STEP 20000] FROM NAMED WINDOW :q1_wz ON STREAM :acc_z [RANGE 60000 STEP 20000] % Query instance Q2 FROM NAMED WINDOW :q2_wx ON STREAM :acc_x [RANGE 60000 STEP 20000] FROM NAMED WINDOW :q2_wy ON STREAM :acc_y [RANGE 60000 STEP 20000]
FROM NAMED WINDOW :q2_wz ON STREAM :acc_z [RANGE 60000 STEP 20000] 

The corresponding WINDOW clauses use the same respective named-window identifiers. Apart from these identifiers, the graph patterns, stream references, window range and step, and result expressions remain unchanged. Because the current Query Registry requires named-window identifiers to match, the two instances are treated as non-reusable and therefore create independent RSP executions. In **E4a**, both instances reference the same physical streams as illustrated above. In **E4b**, the same controlled non-reuse mechanism is used while each query instance additionally references a distinct set of physical streams. As the current query-reuse mechanism requires the named-window identifier to match, these semantically equivalent query instances are treated as non-reusable and therefore result in independent RSP query executions.

This controlled variation avoids introducing differences in computational complexity between the query instances. Consequently, changes in latency, resource consumption, and processing completeness as the number of queries increases primarily reflect the cost of maintaining additional independent query-processing pipelines rather than differences in the computational complexity of the queries themselves.

Two workload configurations are considered to distinguish the effect of query-execution sharing from the effect of stream-acquisition sharing:

1. **Non-reusable query instances over the same streams**: The query instances use different named-window identifiers and therefore cannot reuse an existing query execution under the current Query Registry, while all instances operate over the same underlying physical streams. Heimdall consequently maintains an independent RSP execution for each query instance while physical stream subscription and retrieval remain shared.
2. **Non-reusable query instances over different streams**: The same controlled non-reuse mechanism is applied, but each query instance additionally operates over a different set of physical streams. Consequently, Heimdall maintains both independent RSP executions and independent stream-acquisition paths as the number of active query instances increases.

The first configuration isolates the computational cost of maintaining an increasing number of independent continuous-query executions while keeping physical stream acquisition shared. The second configuration additionally increases the stream-acquisition workload, representing a case in which fewer opportunities for sharing exist. Comparing the two configurations therefore allows us to distinguish the scalability benefit obtained from sharing physical stream acquisition from that obtained through query execution reuse.

For both configurations, we measure processing completeness, latency, CPU utilization, RSS memory consumption, and host-boundary network traffic. Processing completeness measures whether Heimdall keeps pace with the expected physical input during the experimental interval.

## 8. Evaluation Results

This section presents the results of the four experiments described in Section 7. We first evaluate the scalability of the three stream-processing architectures under increasing concurrent client load and subsequently decompose their latency into one-time and recurring operations. We then examine the effect of query and data heterogeneity on latency, resource consumption, and network traffic. Finally, we evaluate the scalability of Heimdall under concurrent non-reusable queries, distinguishing between workloads in which physical stream acquisition remains shared and those in which both query execution and stream acquisition must be performed independently.

### 8.1. E1: Scalability Under an Increasing Number of Clients

#### 8.1.1. Concurrent Execution and Latency

**Figure 8:**
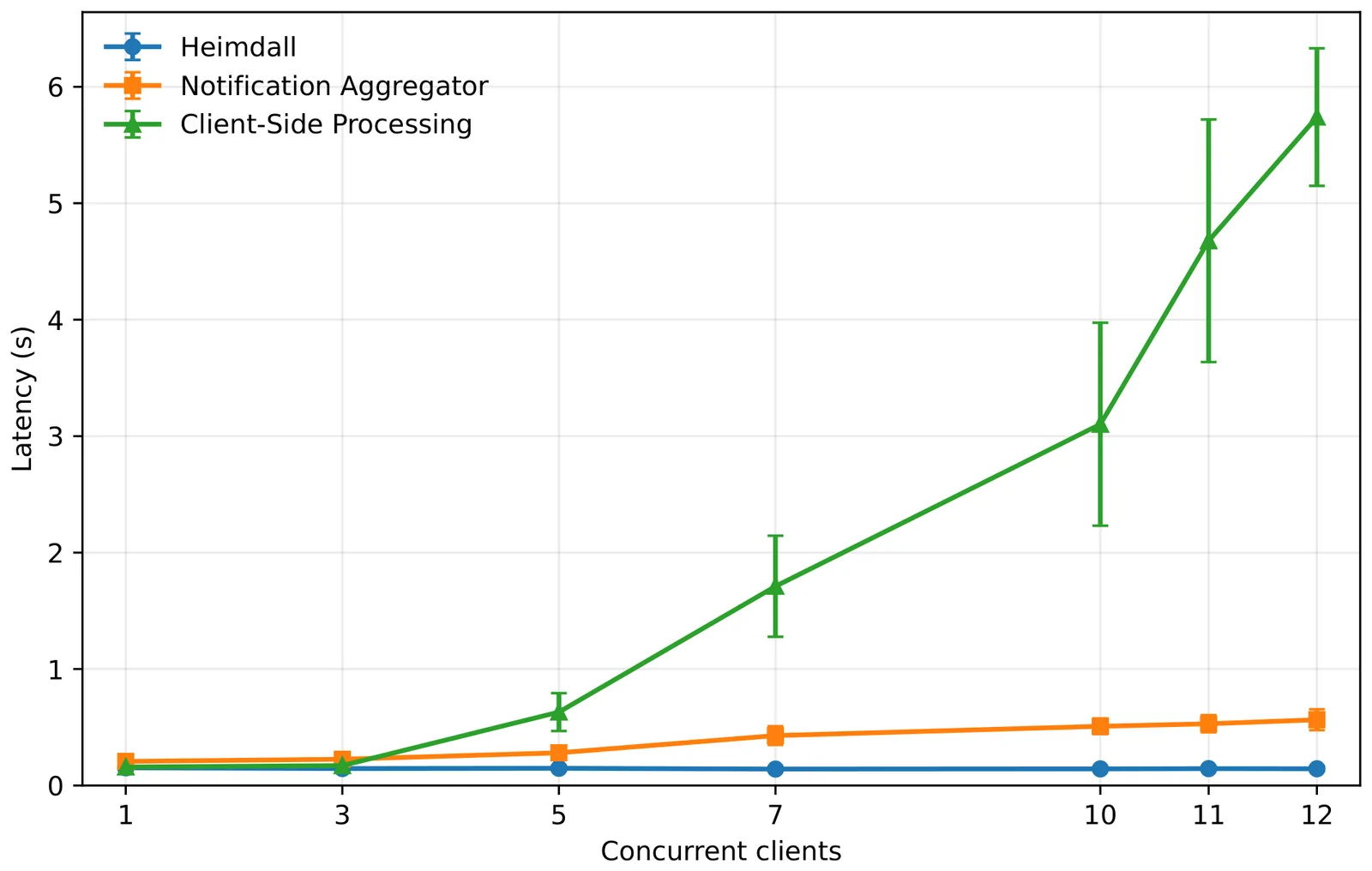
Latency under increasing concurrent client load for Heimdall, the Notification Aggregator, and Client-Side Processing. Error bars indicate standard deviation across successful repetitions. Heimdall maintains nearly constant latency, while the Notification Aggregator shows a moderate increase and Client-Side Processing degrades sharply with additional clients. Client-Side Processing is unstable at 11 and 12 clients, with only 28/30 and 12/30 successful runs, respectively; the plotted values therefore reflect only successful repetitions.

Figure 8 shows how the latency of the three approaches changes as the number of concurrently executing clients increases. At one client, all three approaches exhibit comparable latency with 0.152 s for Heimdall, 0.207 s for the Notification Aggregator, and 0.159 s for Client-Side Processing. The behavior diverges as concurrency increases. Heimdall remains effectively constant across the complete experiment, with latency between 0.141 s and 0.152 s from 1 to 12 clients. The Notification Aggregator shows a gradual increase, reaching 0.565 s at 12 clients. In contrast, Client-Side Processing begins to degrade substantially from five clients onward, increasing from 0.631 s at five clients to 1.711 s at seven clients and 3.102 s at ten clients. Importantly, all 30 Client-Side Processing repetitions still succeeded at 10 clients, showing that the increase in latency precedes outright execution failures.

Table 1 shows where this degradation develops into instability. Heimdall and the Notification Aggregator completed all 30 repetitions at every tested client count. Client-Side Processing also maintained a 100% success rate up to 10 clients, but this dropped to 93.3% at 11 clients and only 40.0% at 12 clients. Consequently, the 4.678 s and 5.740 s latency values shown for 11 and 12 clients in Figure 8 are calculated only from the 28 and 12 successful repetitions, respectively, and should not be interpreted as stable operating points. Heimdall therefore maintains stable latency across the tested client counts, whereas Client-Side Processing becomes unstable beyond 10 clients.

#### 8.1.2. Out-of-Order Event Behavior Under Concurrent Load

**Figure 9:**
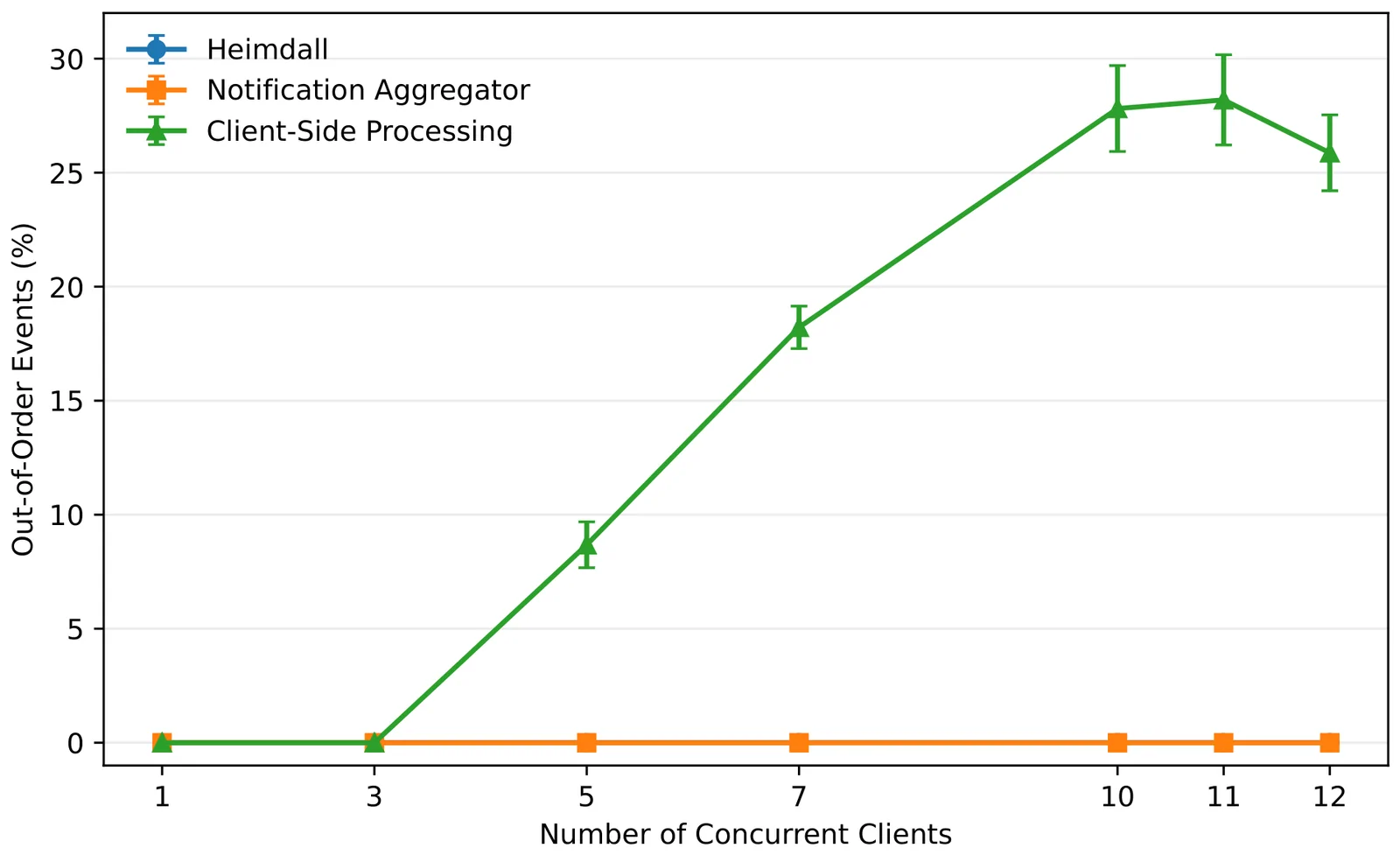
Out-of-order event rate under increasing concurrent client load. Error bars indicate standard deviation across successful repetitions. Heimdall and the Notification Aggregator exhibit no out-of-order events throughout the evaluated range, whereas Client-Side Processing begins to exhibit increasing event reordering from five concurrent clients onward. Client-Side Processing values at 11 and 12 clients are calculated from the 28/30 and 12/30 successful repetitions, respectively.

Figure 9 shows how increasing concurrent load affects the ordering of events observed by the query-processing pipelines. No out-of-order events are observed for Heimdall or the Notification Aggregator throughout the evaluated client range. Client-Side Processing similarly exhibits no event reordering at one and three clients, but out-of-order events emerge from five concurrent clients onward and increase to approximately 28% at ten and eleven clients. The increase in out-of-order events coincides with the latency degradation observed for Client-Side Processing.

#### 8.1.3. Concurrent Resource Consumption

**Figure 10:**
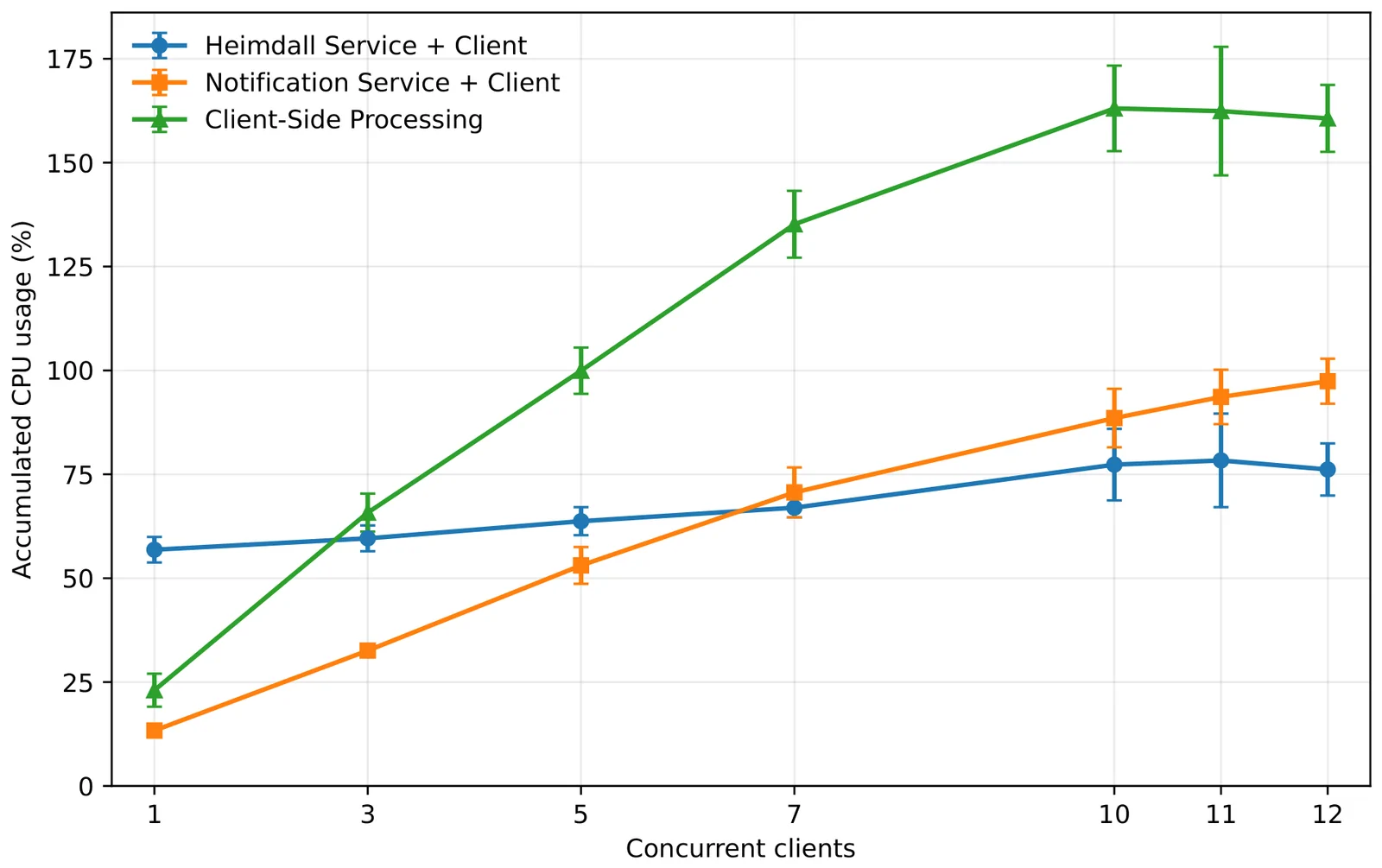
Accumulated CPU usage under increasing concurrent client load. For Heimdall and the Notification Aggregator, the reported CPU usage combines the shared service process with all concurrently executing client processes, and Client-Side Processing contains only the client processes because no shared service is present. Error bars indicate the standard deviation across successful repetitions. Heimdall exhibits substantially slower growth in accumulated CPU usage as concurrency increases, whereas the Notification Aggregator and Client-Side Processing require progressively more client-side computation. Client-Side Processing is unstable at 11 and 12 clients, with only 28/30 and 12/30 successful runs, respectively.

Figure 10 shows the accumulated CPU usage of the complete processing architecture as concurrent demand increases. For Heimdall and the Notification Aggregator, this includes both the shared service and all concurrent client processes, whereas Client-Side Processing consists entirely of client-side processes. Heimdall has the highest accumulated CPU usage at one client (56.86%) because of its fixed service cost, but it grows more slowly with concurrency. At ten clients, accumulated CPU usage is 77.33% for Heimdall, compared with 88.54% for the Notification Aggregator and 163.05% for Client-Side Processing. The Client-Side Processing measurements at 11 and 12 clients reflect only the 28 and 12 successful repetitions and should not be interpreted as stable operating points.

**Figure 11:**
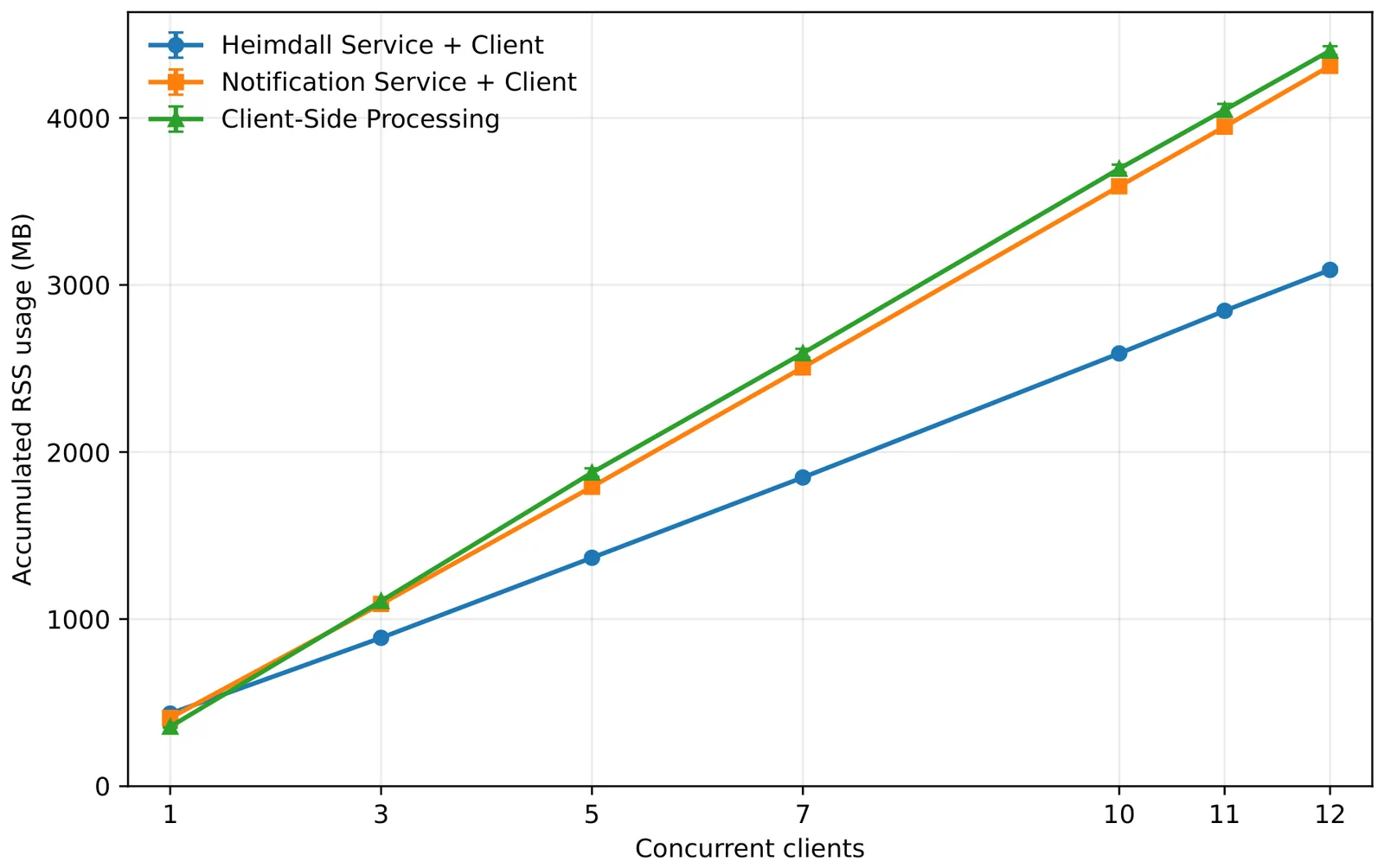
Accumulated RSS memory usage under increasing concurrent client load. For Heimdall and the Notification Aggregator, memory usage combines the shared service process with all concurrently executing client processes, while Client-Side Processing represents the accumulated memory of the client processes alone. Heimdall exhibits a lower rate of memory growth as the number of clients increases, while the Notification Aggregator and Client-Side Processing show similar and substantially higher accumulated memory consumption. Client-Side Processing measurements at 11 and 12 clients correspond only to the successful repetitions.

Figure 11 shows that accumulated RSS also grows more slowly for Heimdall. At 12 clients, Heimdall uses 3090.37 MB, compared with 4311.30 MB for the Notification Aggregator and 4402.33 MB across the successful Client-Side Processing repetitions.

#### 8.1.4. Effect of Scaling on the Network Traffic

**Figure 12:**
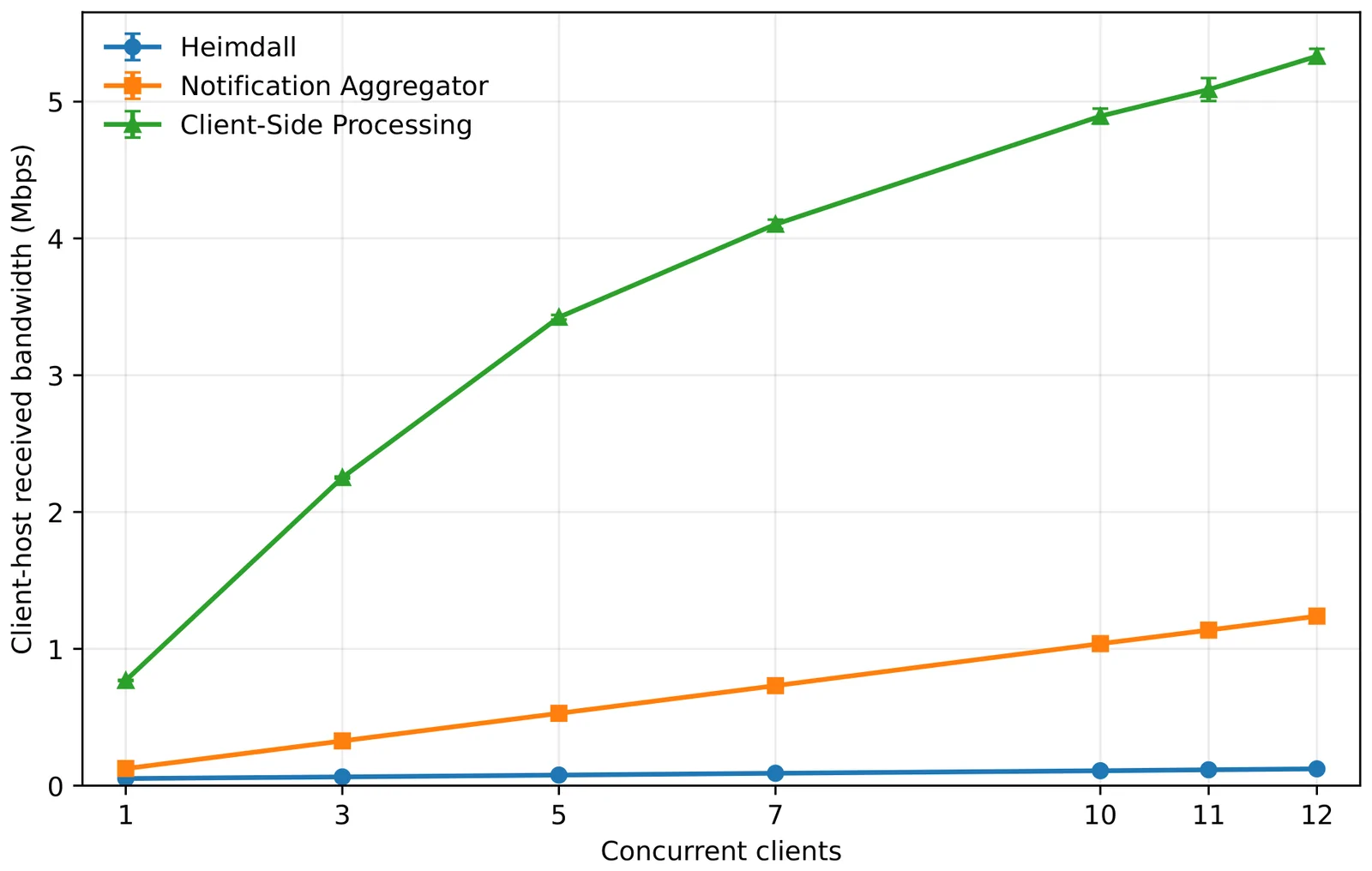
Aggregate bandwidth received by the client host under increasing concurrent client load. Error bars indicate the standard deviation across successful repetitions. Heimdall exhibits only a small increase in received bandwidth as additional clients are introduced, whereas the Notification Aggregator increases approximately with the number of clients, and Client-Side Processing produces substantially higher client-host traffic. Client-Side Processing measurements at 11 and 12 clients correspond only to the successful repetitions.

Figure 12 first shows the aggregate bandwidth received by the host executing the concurrent clients. The difference between the three architectures becomes immediately visible as concurrency increases. Client-host receive bandwidth increases from 0.052 to 0.123 Mbps for Heimdall and from 0.125 to 1.239 Mbps for the Notification Aggregator between 1 and 12 clients. Client-Side Processing reaches 4.894 Mbps at 10 clients, whereas its 11- and 12-client values are based only on successful repetitions.

**Figure 13:**
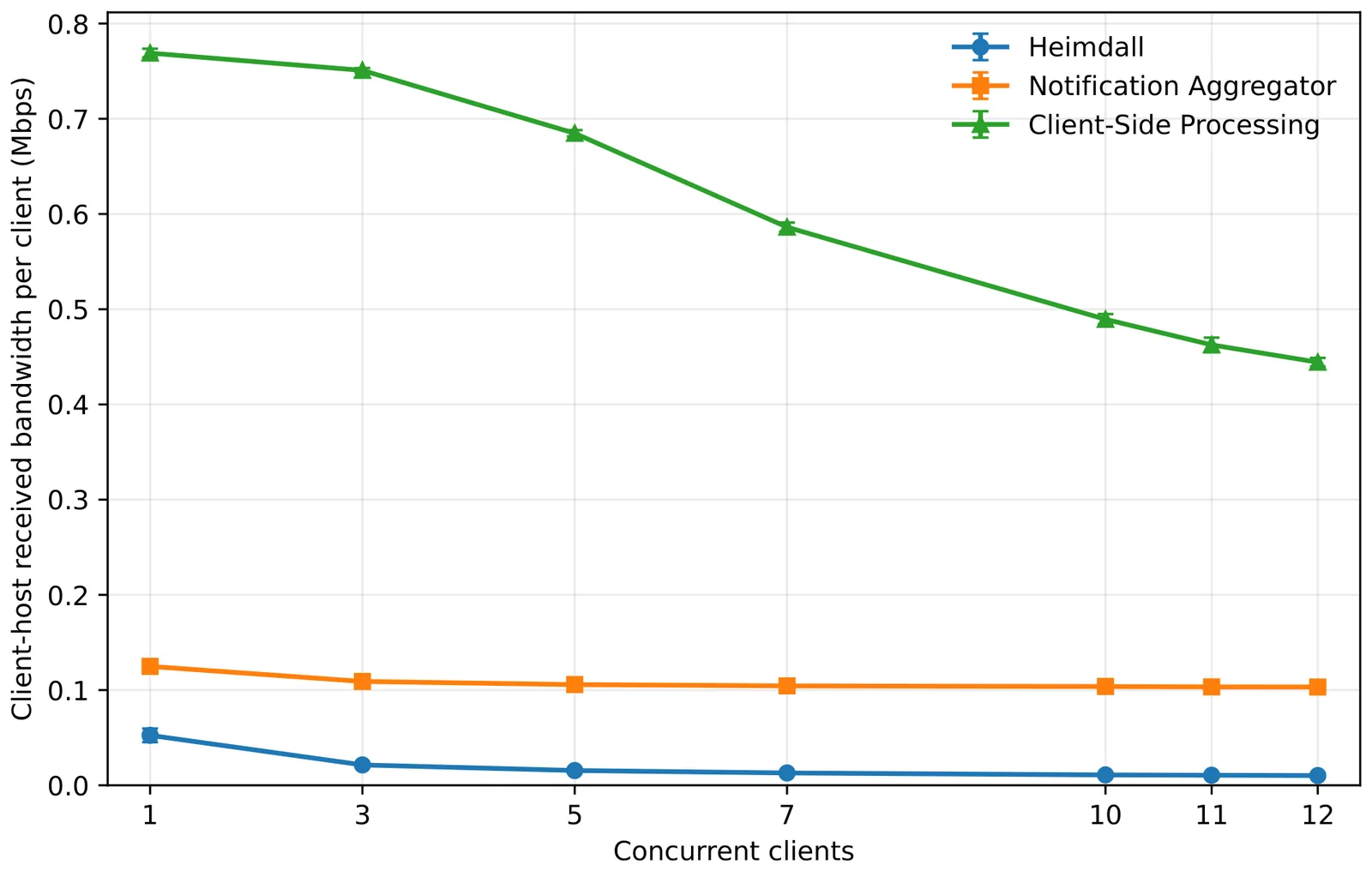
Client-host received bandwidth normalized by the number of concurrent clients. The aggregate received bandwidth of the client host is divided by the number of active clients to illustrate the network cost per client. Heimdall shows a decreasing per-client cost as concurrency increases, while the Notification Aggregator remains approximately constant. The decreasing values for Client-Side Processing at higher concurrency should not be interpreted as improved efficiency, as this architecture simultaneously exhibits increasing latency and becomes unstable at 11 and 12 clients.

Figure 13 shows that per-client receive bandwidth decreases from 0.0525 to 0.0102 Mbps for Heimdall between 1 and 12 clients, while the Notification Aggregator remains close to 0.10 Mbps per client. Client-Side Processing remains substantially higher; its apparent reduction at high concurrency coincides with severe latency degradation and should not be interpreted as improved scalability.

**Figure 14:**
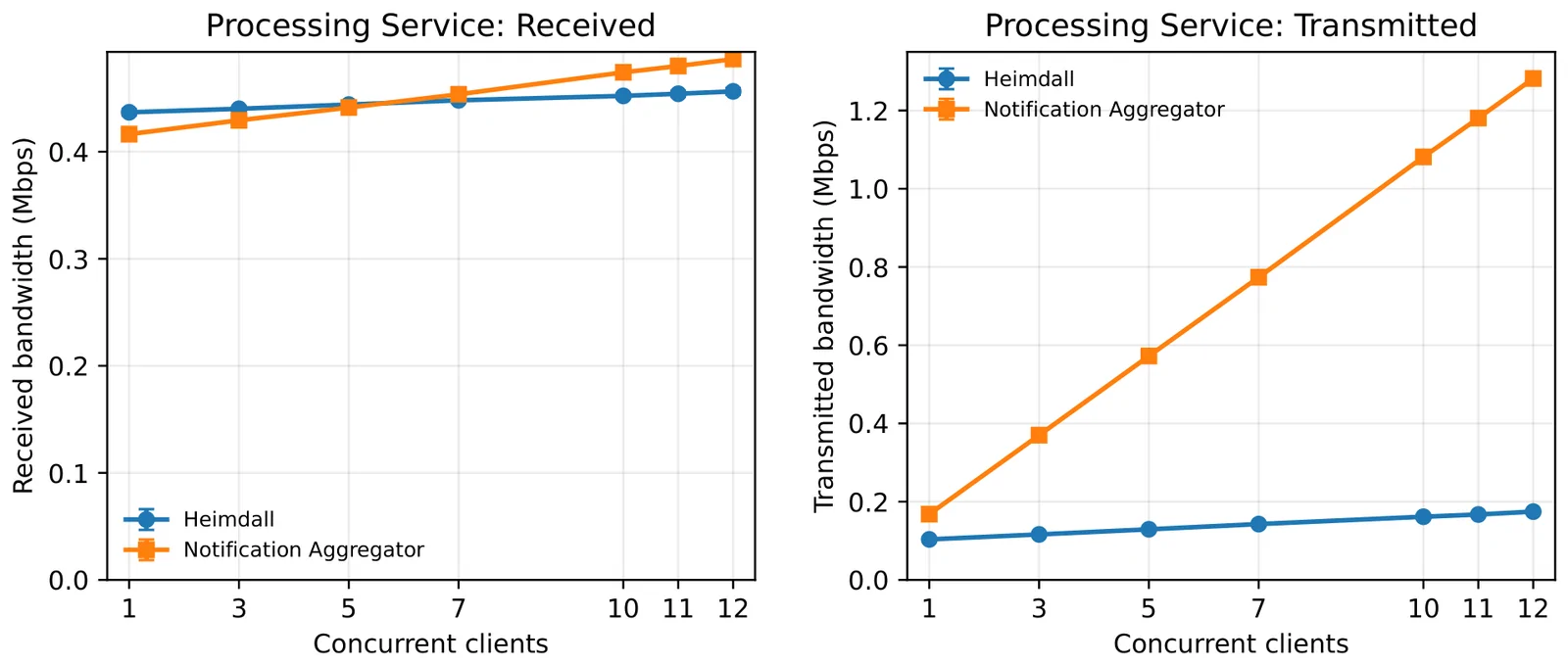
Received and transmitted bandwidth of the shared processing services under increasing concurrent client load. Both Heimdall and the Notification Aggregator maintain nearly constant received bandwidth because stream retrieval is shared independently of the number of clients. Their transmitted bandwidth differs substantially as Heimdall sends only shared query results to the clients, whereas the Notification Aggregator forwards stream events to each client for local processing. It is worth noting that Client-Side Processing is not shown because it has no intermediary processing service.

Figure 14 isolates the network traffic at the intermediary services and reveals where the difference between Heimdall and the Notification Aggregator arises. The left panel shows that their received bandwidth remains almost independent of the number of clients. Heimdall increases only from 0.437 Mbps at one client to 0.456 Mbps at 12 clients, while the Notification Aggregator increases from 0.416 to 0.487 Mbps.

The right panel shows a substantially different pattern for transmitted bandwidth. Heimdall increases only from 0.104 to 0.175 Mbps between 1 and 12 clients because the shared service performs the continuous-query execution and transmits only the resulting query updates. In contrast, the Notification Aggregator increases from 0.168 to 1.282 Mbps because the service must forward the retrieved stream events to every client, where the RSP query is subsequently executed locally.

**Figure 15:**
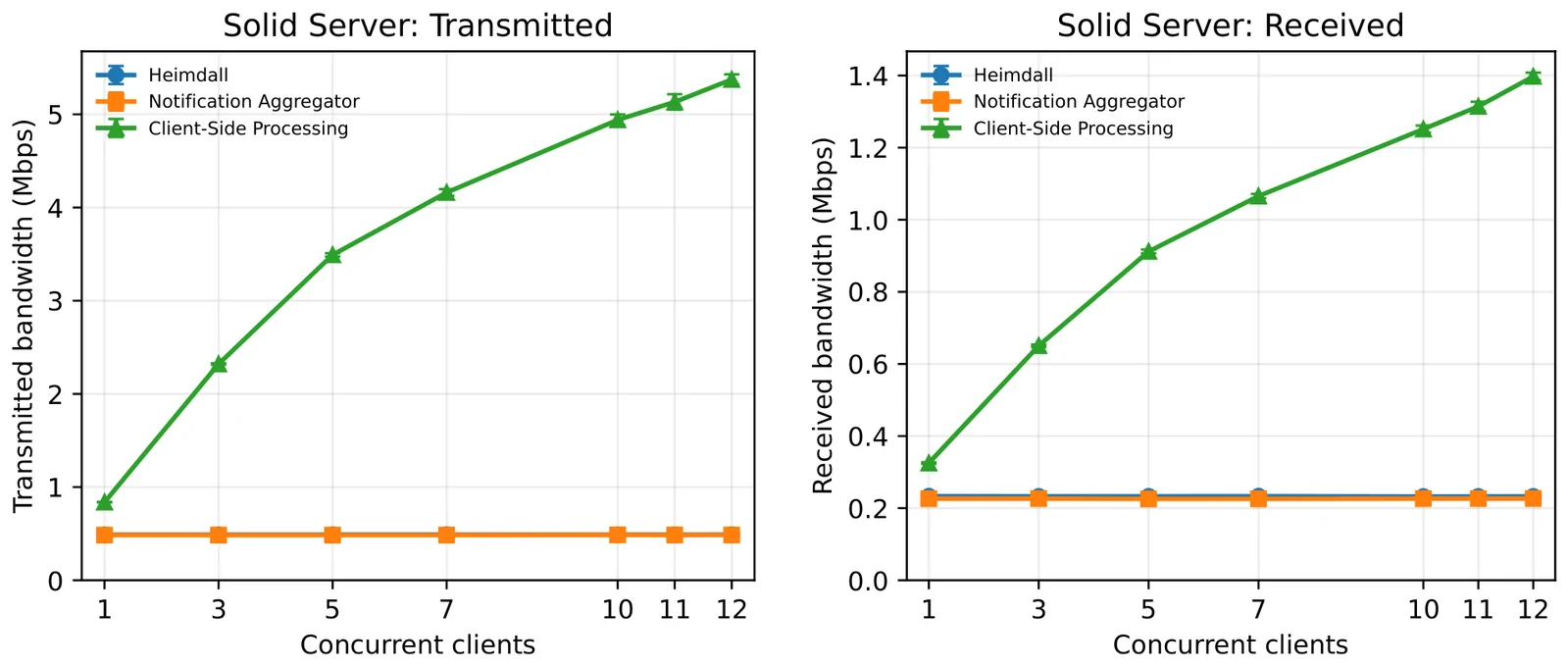
Transmitted and received bandwidth at the Solid server under increasing concurrent client load. Heimdall and the Notification Aggregator maintain nearly constant Solid-server traffic because a shared intermediary retrieves the stream independently of the number of clients. In contrast, Client-Side Processing causes both transmitted and received bandwidth at the Solid server to increase substantially as each client interacts with the server independently. Client-Side Processing measurements at 11 and 12 clients correspond only to successful repetitions.

Finally, Figure 15 shows that Solid-server network traffic remains approximately constant for both intermediary architectures, whereas Client-Side Processing increases substantially with concurrent client load. Solid-server transmitted bandwidth remains approximately 0.49 Mbps for Heimdall and the Notification Aggregator, while Client-Side Processing increases from 0.838 Mbps at 1 client to 4.941 Mbps at 10 clients and reaches 5.374 Mbps among the successful 12-client repetitions. Similarly, received bandwidth remains approximately 0.233 Mbps for Heimdall and 0.226 Mbps for the Notification Aggregator, whereas Client-Side Processing increases from 0.325 Mbps at 1 client to 1.251 Mbps at 10 clients and 1.398 Mbps among the successful 12-client repetitions.

#### 8.1.5. Projected Accumulated Decentralized Resource Consumption

**Figure 16:**
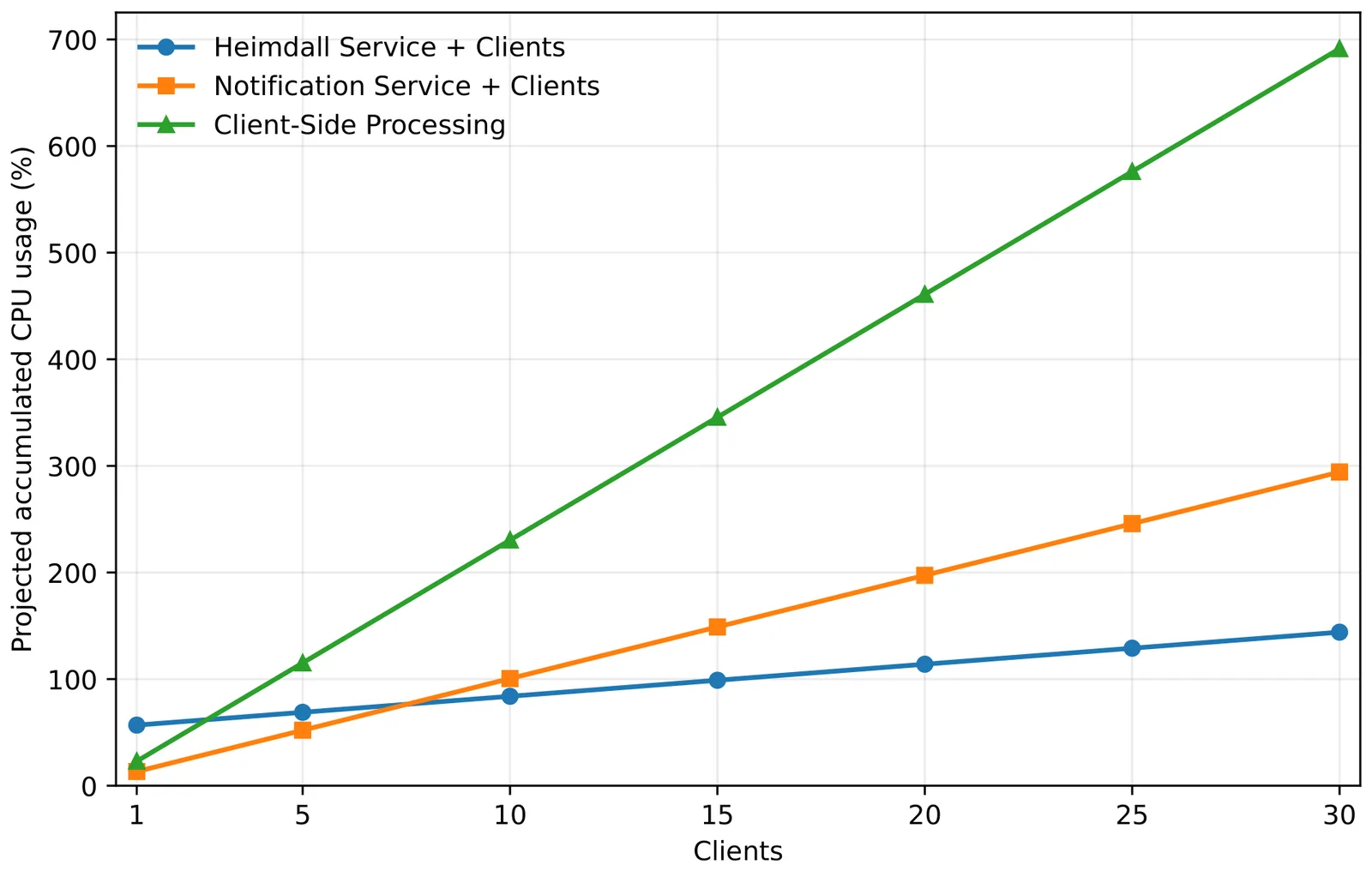
Projected accumulated decentralized CPU consumption as the number of clients increases. The projection is derived from the measured one-client CPU usage of each architecture and assumes that each additional client contributes the same client-side CPU cost, while the shared Heimdall and Notification services are instantiated only once. Accordingly, the projected resource consumption is computed as the shared service cost plus *n* times the measured single-client cost. Heimdall exhibits a substantially lower growth rate because most stream retrieval and continuous-query processing is performed once by the shared service rather than repeated independently by every client.

Figure 16 shows the projected accumulated CPU demand for independently provisioned clients. At one client, Heimdall consists of 53.85% service CPU and 3.01% client CPU, compared with 3.65% service CPU and 9.69% client CPU for the Notification Aggregator and 23.05% client CPU for Client-Side Processing. At 30 clients, the projected accumulated CPU demand reaches approximately 144% for Heimdall, compared with approximately 294% for the Notification Aggregator and 691% for Client-Side Processing.

**Figure 17:**
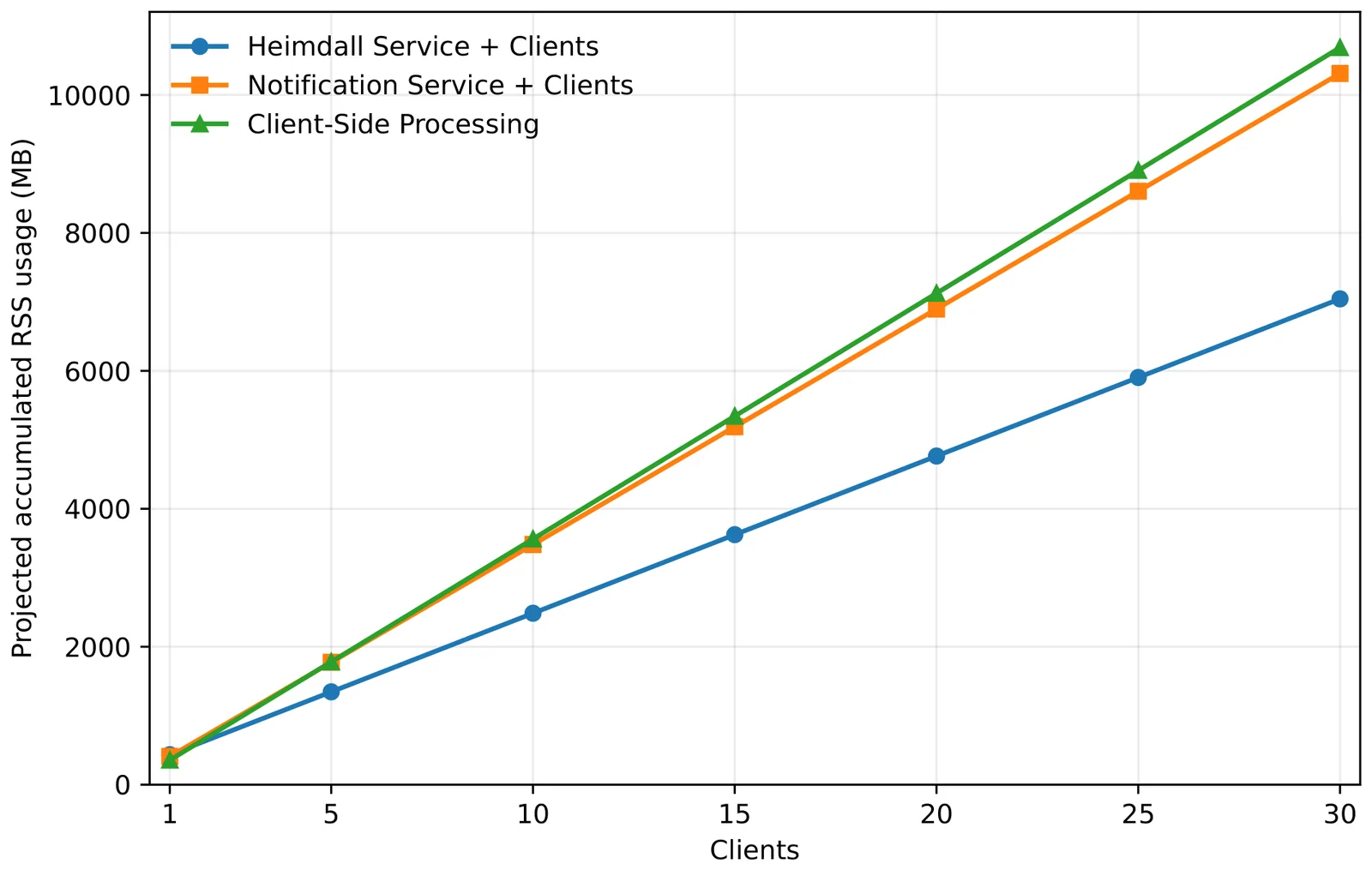
Projected accumulated decentralized RSS memory consumption as the number of clients increases. The projection uses the measured one-client memory footprint and assumes one shared service instance for Heimdall and the Notification Aggregator, together with *n* independent client processes. Heimdall shows a substantially lower growth rate because its client processes have a smaller memory footprint, whereas the Notification Aggregator and Client-Side Processing retain considerably more processing functionality at each client.

Figure 17 shows the corresponding projected accumulated memory demand. The measured one-client RSS footprint is approximately 206.81 MB for the Heimdall service and 227.91 MB for its client, compared with 67.03 MB for the Notification service and 341.53 MB for its client, while Client-Side Processing requires approximately 356.29 MB per client. At 30 clients, the projected accumulated RSS is approximately 7.0 GB for Heimdall, 10.3 GB for the Notification Aggregator, and 10.7 GB for Client-Side Processing.

These projections complement the concurrent measurements by representing accumulated resource demand when clients execute on independently provisioned machines. The projected CPU percentages therefore represent summed CPU demand across decentralized machines rather than utilization of a single physical host.

### 8.2. E2: Latency Decomposition

Table 2 decomposes the latency of the three evaluated stream-processing approaches in the single-client configuration. The measurements distinguish between one-time operations performed during initialization and recurring operations performed while processing the stream.

#### 8.2.1. One-Time Initialization Overheads

The Client-Side Processing approach requires the smallest set of initialization operations because the client interacts directly with the Solid Pod. Its measured initialization operations consist of stream discovery (18.86±3.82 ms) and stream subscription (37.64±5.79 ms).

The Notifications Aggregator additionally requires discovery and authentication of the intermediary service. Service discovery required 25.65±2.34 ms and service authentication required 35.40±6.50 ms. Stream discovery required 25.96±5.81 ms, while the measured stream-subscription operation required 0.24±0.06 ms.

Heimdall introduces the largest number of initialization operations because the client first discovers and authenticates with the service and then registers its continuous query. However, the Heimdall-specific query-management operations themselves introduce little latency. The query reuse check required only 0.19±0.02 ms, query registration required 0.27±0.04 ms, and transmission of the WebSocket query message required 0.64±0.33 ms. The largest individual initialization cost for Heimdall was stream subscription at 85.85 ± 9.16 ms, followed by service authentication at 28.45±3.40 ms.

#### 8.2.2. Recurring Stream-Processing Overheads

Among the recurring operations, query evaluation contributes the largest measured latency for all three approaches. Mean query-evaluation latency is 76.54±12.42 ms for Client-Side Processing, 120.71±15.84 ms for the Notification Aggregator, and 51.52 ± 14.77 ms for Heimdall. Event retrieval is the next largest recurring component, while parsing, timestamp extraction, insertion into the RSP engine, and result dispatch remain comparatively small.

For recurring operations, Table 2 reports both mean ± standard deviation and median [$Q_{1}$, $Q_{3}$]. Most of the lower-level operations, such as parsing and insertion into the RSP engine, show similar mean and median values and relatively narrow interquartile ranges. Greater variability is observed for query evaluation. For example, the Notifications Aggregator has a mean query-evaluation latency of 120.71 ms compared with a median of 109.75 ms, while Heimdall has a mean of 51.52 ms compared with a median of 45.65 ms. Reporting both statistics therefore distinguishes the typical observed latency from variability caused by higher-latency observations.

### 8.3. E3: Query and Data Heterogeneity

E3 examines whether the behavior of the three stream-processing architectures changes when the query and/or the underlying input data are varied. E3 compares a single-query client under the three architectures for the same-query/same-data, different-query/same-data, and different-query/different-data workloads.

#### 8.3.1. Effect of Heterogeneity on Latency

Table 3 compares the first-result latency across the three query and data workload scenarios. The tested variations have only a limited effect on the latency of any of the three architectures. Heimdall achieves 160.45±30.29 ms for the same-query/same-data workload, 157.54±11.55 ms for different queries over the same data, and 158.71±12.59 ms when both the query and data differ. Relative to the same-query/same-data workload, the latter two scenarios change the mean latency by only −1.81% and −1.08%, respectively. The Notification Aggregator exhibits similarly stable behavior, with mean latency ranging from 204.31 to 206.27 ms across the three scenarios. Client-Side Processing shows slightly greater variation, increasing from 156.85 ms for the same-query/same-data workload to 164.82 ms for different queries over the same data and 162.59 ms when both the query and data differ. Its largest increase relative to the homogeneous workload is 5.08%. Overall, first-result latency remains largely stable across the three E3 workload scenarios.

#### 8.3.2. Effect of Heterogeneity on Resource and Network Usage

Table 4 shows that the tested query and data variations have little effect on resource consumption within each architecture.

Resource consumption remains stable within each architecture across the three workloads. Heimdall client CPU stays at 2.66–2.69% and service CPU at 57.98–58.21%; the Notification Aggregator client CPU remains at 10.41–10.78%, while Client-Side Processing remains at 22.83–23.85%. RSS varies similarly little within each architecture. These architecture-specific resource profiles remain largely unchanged across the three E3 workload scenarios.

Table 5 shows that network traffic is also largely invariant across the tested workload scenarios. Heimdall client receive bandwidth remains between 0.026 and 0.027 Mbps, compared with approximately 0.125 Mbps for the Notification Aggregator and 0.780–0.782 Mbps for Client-Side Processing. Solid-server transmitted bandwidth similarly remains approximately 0.508 Mbps for Heimdall and 0.504 Mbps for the Notification Aggregator, whereas Client-Side Processing requires approximately 0.857–0.858 Mbps.

At the intermediary service, Heimdall transmits approximately 0.084 Mbps in every scenario, while the Notification Aggregator transmits approximately 0.176 Mbps.

Overall, the tested query and data heterogeneity introduces substantially less variation than the differences caused by the architectural placement of stream retrieval and continuous-query execution.

### 8.4. E4: Scalability Under Concurrent Non-Reusable Queries

#### 8.4.1. **E4a**: Non-Reusable Query Executions over Shared Streams

E4a increases the number of independent query executions from 1 to 40 while retaining shared acquisition of the same three physical streams.

#### Processing Completeness

Table 6 shows that physical-stream processing remains highly complete through 30 concurrent independent query executions under the evaluated workload. All repetitions are complete at 1, 20, and 25 queries, while 27 of 30 repetitions are complete at 10 queries and 28 of 30 at 30 queries. Mean input completeness therefore remains at or above 96.42% through 30 queries.

A sustained loss of processing completeness appears at 35 queries, where none of the 30 repetitions retrieve all 720 expected observations and mean input completeness decreases to 78.50%. At 40 queries, 4 of 30 repetitions are complete and mean completeness increases to 86.75%. This non-monotonic behavior indicates an unstable high-load region rather than a smooth reduction in processing throughput.

Fan-out correctness remains 100% at every evaluated query count. Thus, every successfully retrieved physical observation is inserted into all corresponding independent query executions. The incomplete runs at higher query counts therefore arise before this internal fan-out step rather than from selective loss while observations are distributed among query executions.

#### Latency, Event Ordering, and Resource Consumption

**Figure 18:**
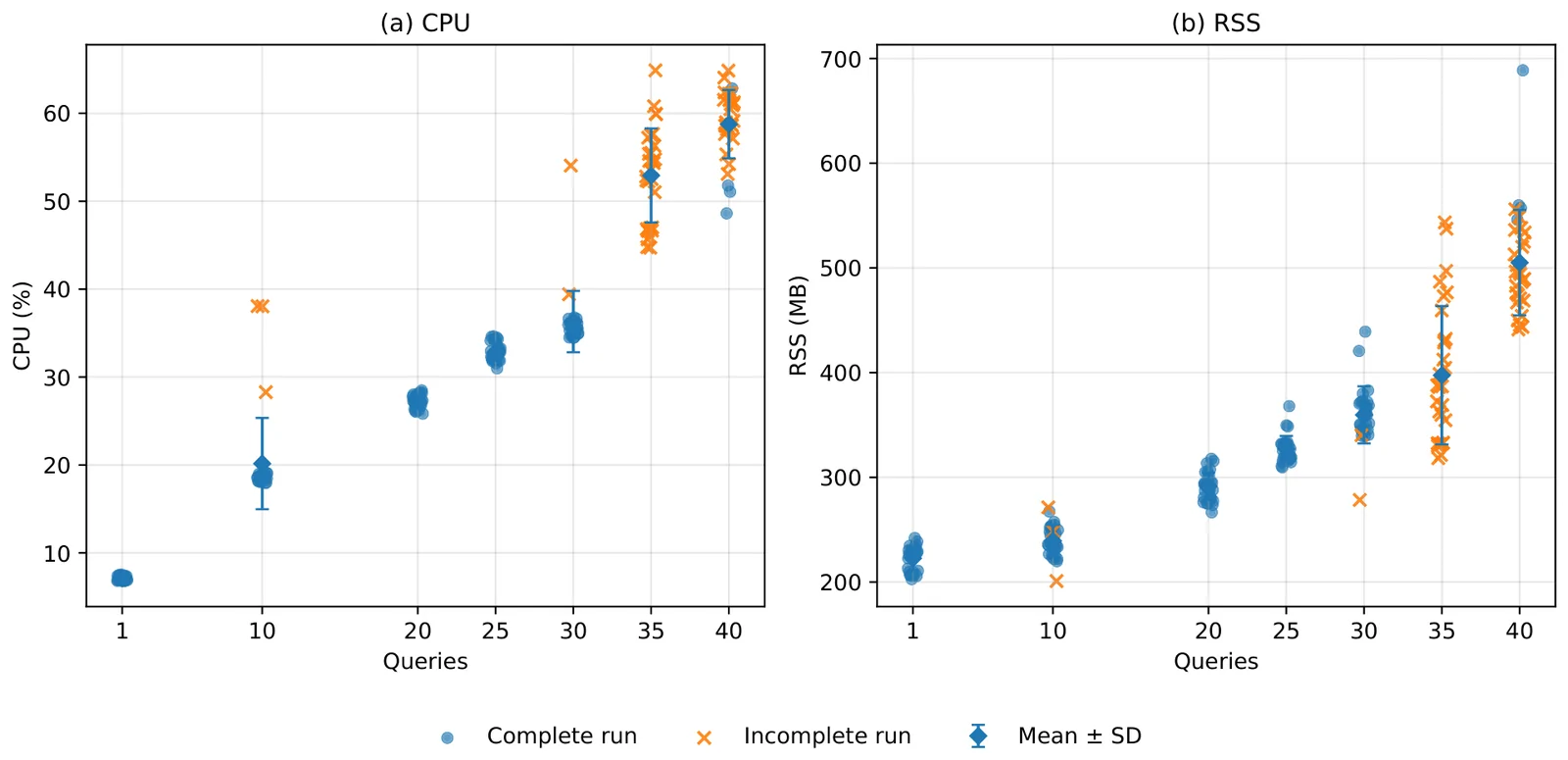
Heimdall service resource consumption under an increasing number of concurrent independent query executions over shared physical streams with (**a**) mean CPU utilization and (**b**) mean RSS memory consumption. Individual markers represent retained repetitions, distinguishing complete from incomplete runs, while diamonds and error bars indicate the mean and standard deviation across all 30 retained repetitions.

Figure 18 shows that the computational cost of maintaining independent query executions increases with the concurrent query workload even though physical-stream acquisition remains shared. Mean CPU utilization rises from 7.12±0.23% for a single query to 32.84±1.01% at 25 queries, 36.31±3.49% at 30 queries, and 58.76±3.89% at 40 queries. RSS memory consumption similarly increases from 222.65±11.61 MB at 1 query to 327.09±12.39 MB at 25 queries, 359.63±27.24 MB at 30 queries, and 505.05±50.38 MB at 40 queries. The increasing CPU and memory requirements show that sharing physical-stream acquisition does not eliminate the computational cost of maintaining additional independent query executions.

The run-level measurements further show that the larger variability at higher query counts reflects genuine run-to-run variation in the high-load region, with incomplete repetitions contributing several of the more extreme CPU and memory measurements.

**Figure 19:**
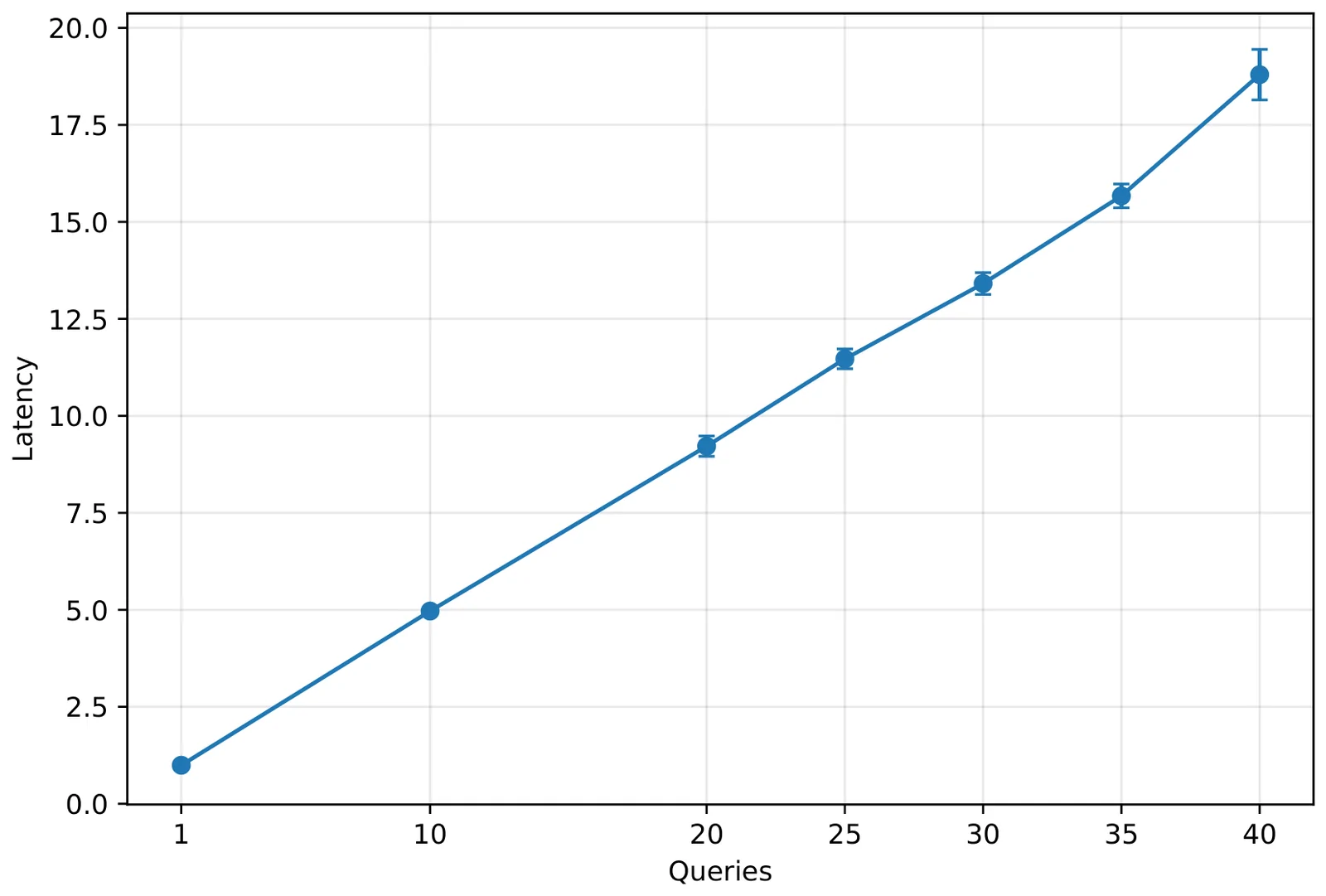
Adjusted first-result latency of Heimdall under an increasing number of concurrent independent query executions over shared physical streams. Error bars indicate the standard deviation across the 30 retained repetitions.

Figure 19 shows that adjusted first-result latency increases consistently with the number of independent query executions. Mean latency rises from 0.993±0.085 s for a single query to 11.469±0.256 s at 25 queries, 13.410±0.281 s at 30 queries, and 18.794 ± 0.651 s at 40 queries. The relatively small standard deviations indicate that this increase is reproducible across repetitions. Notably, latency increases substantially before sustained losses in processing completeness occur, and, at 25 queries, all repetitions still process the complete physical input despite an adjusted latency of approximately 11.5 s.

Table 7 shows that temporal disorder remains limited through 30 concurrent independent query executions. The out-of-order proportion is 0.02% for a single query and remains below 4% through 30 queries, although the progression is not strictly monotonic. Mean lateness among out-of-order observations remains below 1 s over this range, with a maximum observed lateness of 3.26 s.

A stronger increase appears in the high-load configurations. At 35 and 40 queries, 5.80% and 6.55% of classified observations are out of order, respectively, while mean lateness increases to approximately 3 s. Nevertheless, all out-of-order observations remain within the configured 30 s tolerance throughout the evaluated range.

#### Network Behavior

**Figure 20:**
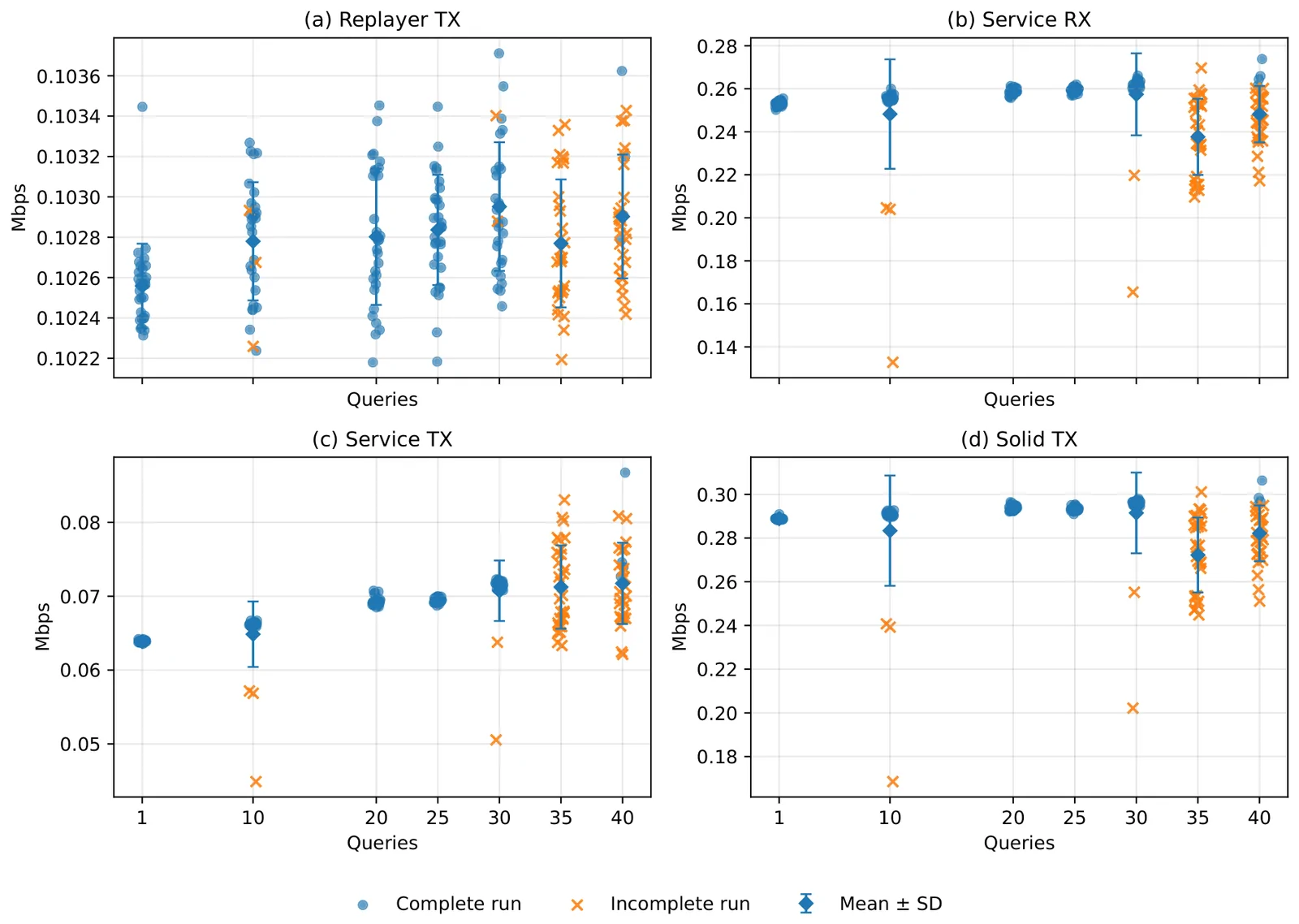
Host-boundary network behavior of Heimdall under an increasing number of concurrent independent query executions over shared physical streams with (**a**) replayer transmission, (**b**) service reception, (**c**) service transmission, and (**d**) Solid-server transmission. Individual markers represent retained repetitions, distinguishing complete from incomplete runs, while diamonds and error bars indicate the mean and standard deviation across all 30 retained repetitions.

Figure 20 shows that increasing the number of independent query executions does not produce a corresponding increase in physical-stream network traffic. Replayer transmission remains effectively constant, from 0.1026±0.0002 Mbps at 1 query to 0.1029±0.0003 Mbps at 40 queries. Service reception changes from 0.2530±0.0012 to 0.2482±0.0131 Mbps, while Solid-server transmission changes from 0.2889±0.0005 to 0.2822±0.0128 Mbps. Service transmission increases only modestly, from 0.0639±0.0001 to 0.0717±0.0055 Mbps.

The run-level measurements additionally show that the increased variability in service reception and Solid-server transmission at higher query counts is associated primarily with incomplete repetitions, whereas replayer transmission remains tightly clustered across the evaluated workload.

The lower service-reception and Solid-transmission values observed in the high-load region coincide with incomplete physical-input processing rather than with a multiplication of stream acquisition. Thus, while CPU usage, memory consumption, and query latency increase as additional independent query executions are maintained, shared physical acquisition prevents network traffic from scaling proportionally with the number of queries.

#### 8.4.2. **E4b**: Non-Reusable Query Executions over Different Streams

#### Processing Completeness

In the different-stream configuration, processing remains complete for 1 and 3 concurrent independent queries, with all 30 retained repetitions retrieving the complete prescribed physical input. Degradation first appears at 5 queries, where only 9 of 30 repetitions retrieve the complete input, although mean input completeness remains 95.17%. At seven queries, none of the repetitions are complete, and mean completeness decreases to 74.59%. At 10 queries, only 1 of 30 repetitions is complete, and mean completeness decreases further to 56.34%.

Despite this reduction in physical-input completeness, retrieval-to-insertion correctness remains 100% at every evaluated query count. Thus, once an observation is successfully retrieved by Heimdall, it is inserted into the corresponding RSP execution without loss. Because **E4b** simultaneously increases the publication, storage, retrieval, and query-processing workload, the experiment does not isolate the contribution of the replayer, Solid server, and Heimdall retrieval process to the observed loss of physical input.

Table 8 summarizes the processing completeness and retrieval-to-insertion correctness for the different-stream configuration.

#### Latency, Event Ordering, and Resource Consumption

**Figure 21:**
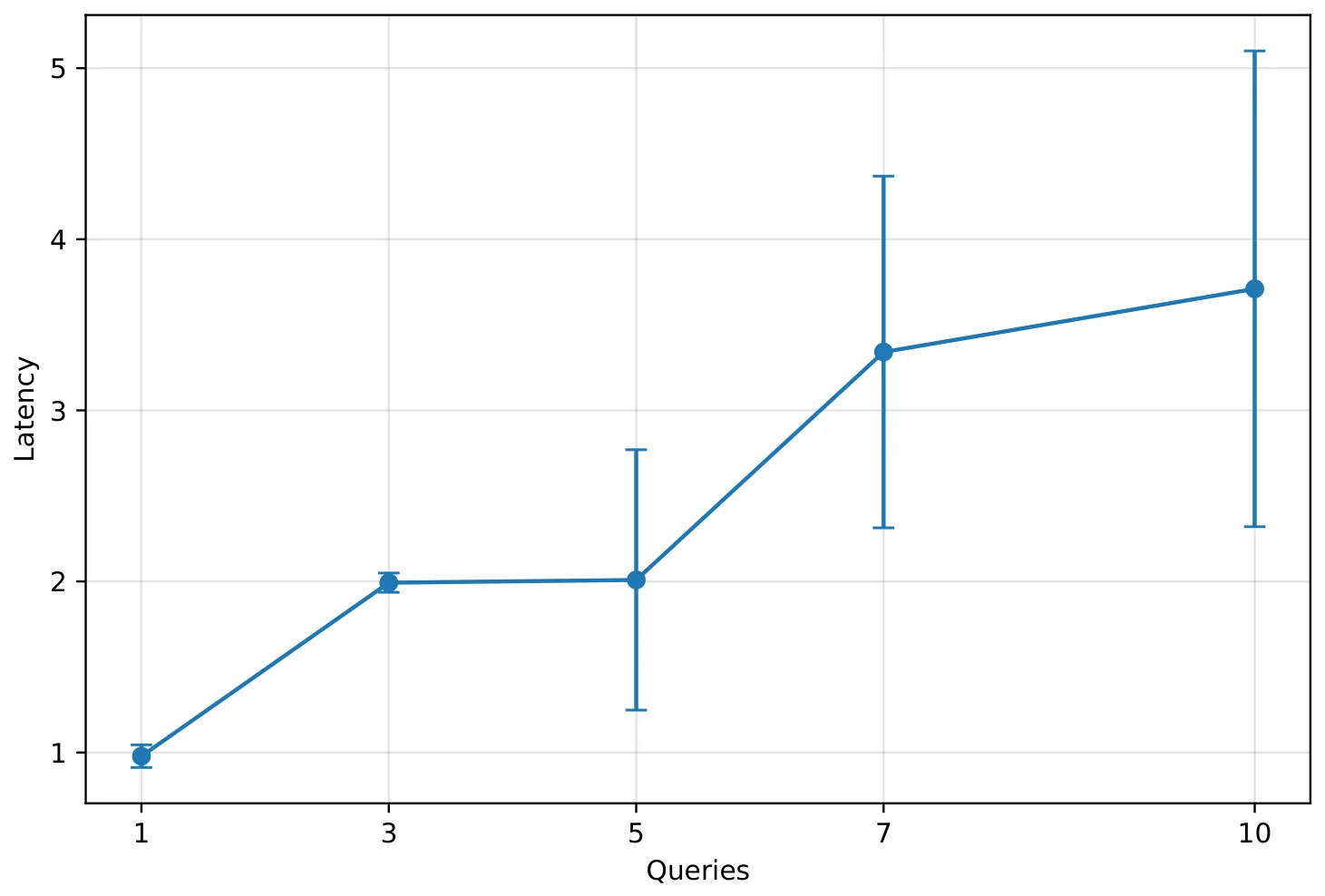
Adjusted first-result latency of Heimdall under an increasing number of concurrent independent queries over different physical streams. Error bars indicate the standard deviation across the 30 retained repetitions.

Figure 21 shows that adjusted first-result latency increases as the number of concurrent independent query-stream groups grows. Mean latency rises from 0.979±0.066 s for one query to 1.993±0.056 s at three queries, 2.009±0.761 s at five queries, 3.341±1.028 s at seven queries, and 3.710±1.391 s at ten queries. The increase is not strictly proportional to query count, but both the mean latency and its variability increase in the higher-load configurations. The larger variability from five queries onward coincides with the onset of incomplete physical-input processing.

**Figure 22:**
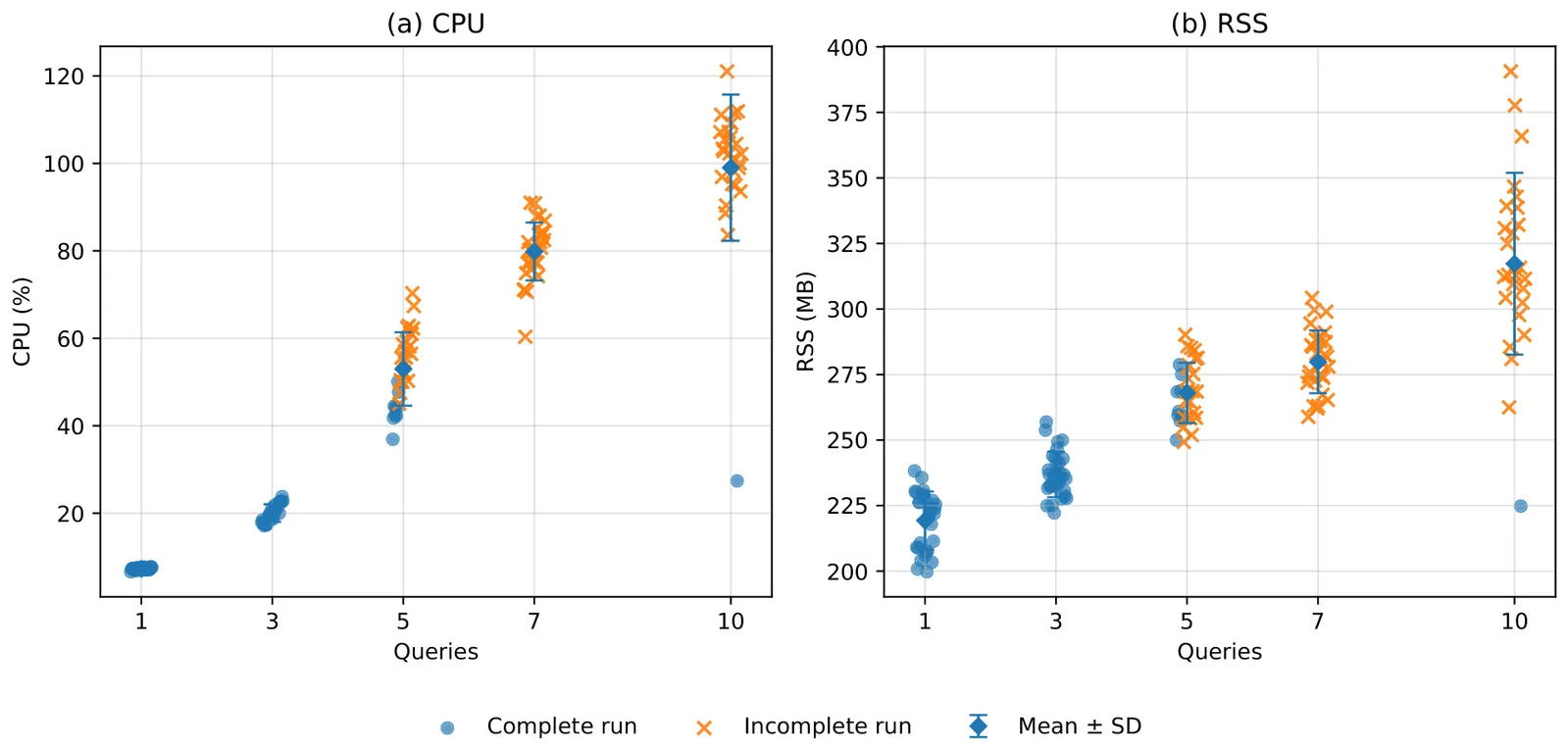
Heimdall service resource consumption under an increasing number of concurrent independent queries over different physical streams with (**a**) mean CPU utilization and (**b**) mean RSS memory consumption. Individual markers represent retained repetitions, distinguishing complete from incomplete runs, while diamonds and error bars indicate the mean and standard deviation across the retained full-duration service traces.

A stronger effect is observed in the resource consumption of the Heimdall service process, shown in Figure 22. Mean CPU utilization rises from 7.30±0.27% at one query to 20.03±2.00% at three queries, 52.98±8.40% at five queries, 79.85±6.62% at seven queries, and 99.02±16.71% at ten queries. RSS memory consumption increases from 219.31 ± 11.08 MB at one query to 267.99±11.54 MB at five queries and 317.28±34.69 MB at ten queries.

For the resource-consumption aggregates, only service traces covering at least 100 s of the experimental period are included. This retains all 30 repetitions at one, three, and five queries, 29 repetitions at seven queries, and 26 repetitions at ten queries. Incomplete runs are retained when the service itself remains active for the required duration. The increase in CPU utilization therefore reflects the resource demand of the high-load operating region rather than being calculated only from complete runs.

The increasing number of independently acquired physical streams also has a substantial effect on temporal ordering. Table 9 shows that no out-of-order observations are recorded for a single query-stream group, whereas the proportion increases to 20.92% at three queries and 36.92% at five queries. Mean lateness among out-of-order observations similarly increases from 2.45±2.59 s at three queries to 5.44±5.48 s at five queries.

The configured 30 s tolerance is first exceeded at five queries, where maximum lateness reaches 44.34 s, although 99.77% of out-of-order observations remain within the configured bound. At seven queries, the out-of-order proportion increases to 40.58%, with mean lateness of 6.89±6.99 s and maximum lateness of 53.63 s. At ten queries, 41.18% of classified observations are out of order, mean lateness increases to 8.31±8.54 s, and 97.10% of out-of-order observations remain within the configured tolerance.

#### Network Behavior

**Figure 23:**
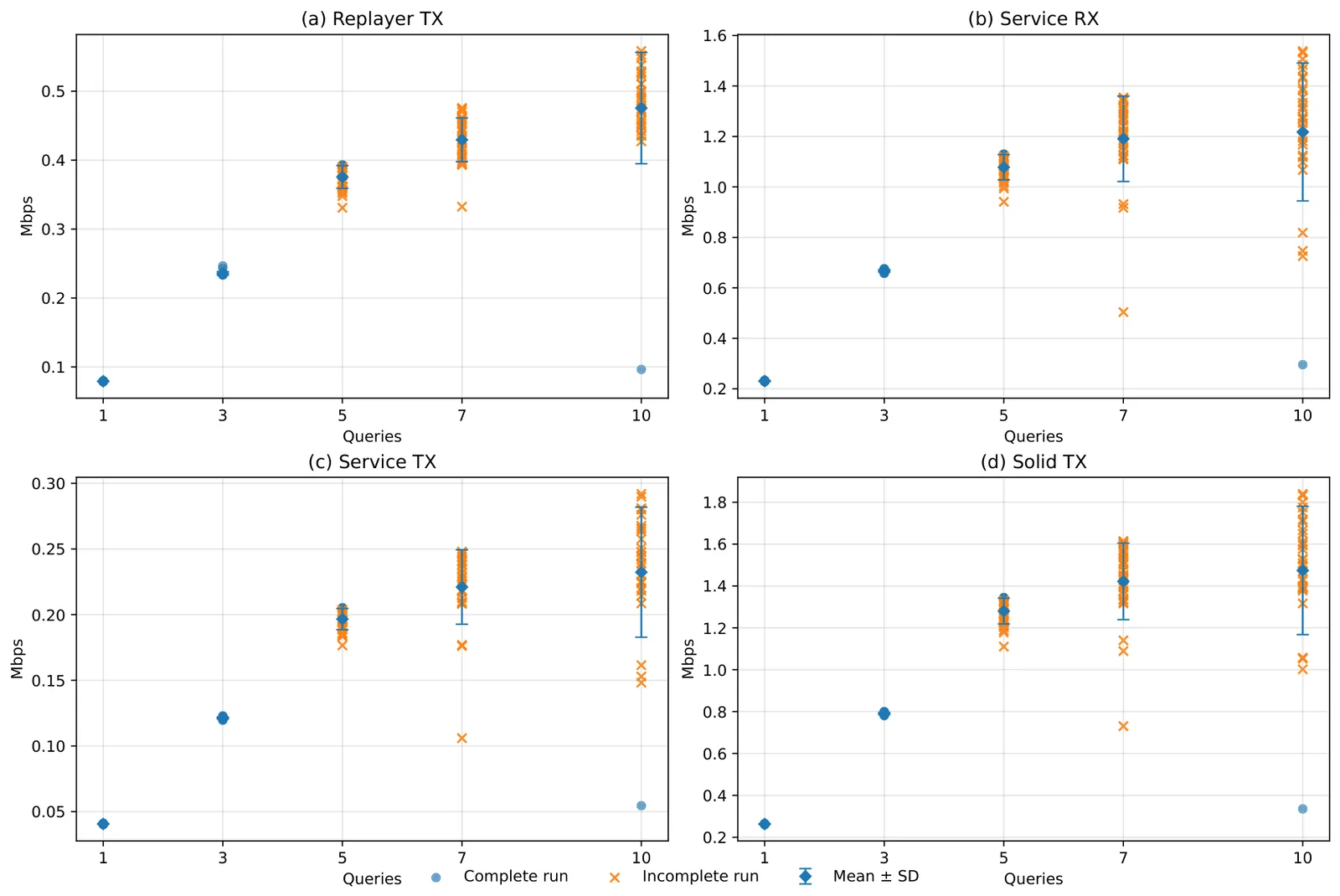
Host-boundary network behavior under an increasing number of concurrent independent queries over different physical streams, (**a**) replayer transmission, (**b**) Heimdall service reception, (**c**) Heimdall service transmission, and (**d**) Solid-server transmission. Error bars indicate standard deviation across repetitions.

Figure 23 shows that network traffic increases substantially when each independent query introduces its own physical streams. From one to five queries, replayer transmission increases from 0.0791±0.0003 to 0.3756±0.0165 Mbps, Heimdall service reception from 0.2309±0.0013 to 1.0780±0.0500 Mbps, service transmission from 0.0406±0.0002 to 0.1966±0.0081 Mbps, and Solid-server transmission from 0.2629±0.0011 to 1.2802±0.0617 Mbps. Unlike **E4a**, each additional query in **E4b** introduces three additional physical streams, so publication and retrieval traffic increase with query count.

The increase becomes less pronounced at seven and ten queries. At ten queries, replayer transmission reaches 0.4755±0.0808 Mbps, service reception 1.2176±0.2731 Mbps, service transmission 0.2323±0.0495 Mbps, and Solid-server transmission 1.4740±0.3062 Mbps. This flattening coincides with the substantial reduction in physical-input completeness and therefore should not be interpreted as improved network efficiency. The increasing standard deviations at the higher query counts similarly reflect greater run-to-run variability in the high-load region.

Overall, **E4b** maintains complete physical-input processing through three concurrent independent queries. Degradation begins at five queries, where mean completeness remains high but only 9 of 30 repetitions process the complete prescribed input, and becomes substantial at seven and ten queries. Retrieval-to-insertion correctness nevertheless remains 100% throughout. Compared with **E4a**, independently increasing physical-stream acquisition results in substantially earlier loss of processing completeness and greater temporal disorder. In particular, all out-of-order observations in **E4a** remain within the configured 30 s tolerance through 40 queries, whereas **E4b** first exceeds this tolerance at 5 queries.

## 9. Discussion

This section interprets the evaluation results in relation to the two research questions. We first discuss how sharing stream acquisition and continuous-query execution affect scalability under increasing client load. We then examine how these benefits change as opportunities for query and data reuse decrease. Finally, we discuss the architectural limitations of Heimdall and the scope of the experimental evaluation.

### 9.1. Interpretation of the Evaluation Results

#### 9.1.1. RQ1: Effect of Sharing Under Increasing Client Load

The results support the hypothesis associated with Research Question 1. The principal advantage of Heimdall does not arise from substantially accelerating an isolated continuous query, as the three architectures exhibit comparable latency at low concurrency. Instead, the advantage emerges as additional clients request the same computation. Client-Side Processing duplicates both stream acquisition and query execution for every client, while the Notification Aggregator shares stream acquisition but retains an independent RSP execution at each client. Heimdall shares both operations when the registered queries are reuse-compatible. Consequently, increasing the number of consumers introduces substantially less duplicated processing and communication in Heimdall.

This distinction is reflected consistently across the latency, resource-consumption, and network measurements. Heimdall maintains approximately stable latency across the evaluated client counts, whereas Client-Side Processing exhibits increasing latency before becoming unstable at the highest tested loads. The accumulated CPU and memory measurements similarly show that architectures retaining more processing at each client incur greater growth as clients are added. The direct concurrent measurements include contention, as all clients’ processes execute on the same physical machine. However, in a decentralized deployment, clients may execute on independently provisioned machines which make the decentralized-client projection usage more representative of the accumulated architectural resource demand without the client-host contention. However, it is important to note that this remains a projection, as we did not execute each client on a separate physical machine, which is left for future work. The network measurements further separate the effects of stream-acquisition sharing and query-execution sharing, as both intermediary architectures prevent Solid server’s traffic from increasing proportionally with the number of clients, but Heimdall additionally avoids forwarding the complete input stream to every client. These results indicate that stream-acquisition sharing primarily protects the decentralized data source, while sharing query execution additionally reduces client-side computation and communication.

**E2** clarifies the cost associated with this sharing. Heimdall introduces additional initialization operations, including service discovery, authentication, query-reuse checking, and query registration. However, the Heimdall-specific query-management operations are small relative to the recurring stream-processing operations, and they are incurred only when establishing the continuous query. The architectural trade-off is therefore a higher initialization complexity and overhead in exchange for avoiding repeated acquisition and execution throughout the lifetime of the query. This trade-off becomes increasingly favorable as either the query lifetime or the number of consumers increases.

#### 9.1.2. RQ2: Effect of Query and Data Overlap

**E3** shows that query and data heterogeneity alone do not substantially change the per-query performance characteristics of the three architectures under the controlled single-client workload. The tested query variants produce similar latency, resource consumption, and network traffic within each architecture. The dominant differences therefore arise from where stream acquisition and query execution are placed rather than from the relatively small differences between the evaluated query-selection patterns themselves. This result is important because it separates the intrinsic cost of the tested heterogeneous queries from the scalability effects that arise when many such queries execute concurrently.

**E4** demonstrates that the degree of sharing becomes consequential once independent queries execute concurrently. In **E4a**, query executions cannot be reused, but the underlying physical streams remain shared. Heimdall therefore incurs an increasing computational and memory cost for maintaining independent RSP pipelines while avoiding duplicated physical-stream acquisition. Under the evaluated workload, physical-input processing remains highly complete through 30 concurrent queries, with mean completeness remaining at or above 96.42% over this range. A sustained degradation appears at 35 queries, where none of the retained repetitions process the complete physical input. Latency and resource consumption increase substantially before this sustained loss of completeness occurs. This shows that sharing stream acquisition remains beneficial even when query-execution reuse is unavailable but does not remove the computational cost of maintaining independent continuous queries.

The contrast with **E4b** is substantially stronger. When each independent query also operates over different physical streams, both the number of RSP executions and the physical-stream acquisition workload increase for Heimdall. Complete processing is maintained for all retained repetitions only through three concurrent queries. At five queries, mean input completeness remains high at 95.17%, but only 9 of 30 repetitions process the complete prescribed input. At seven queries, none of the retained repetitions are complete, and mean completeness decreases to 74.59%, while at ten queries mean completeness decreases further to 56.34%.

CPU utilization, publication and retrieval traffic, and event-time disorder also increase substantially more rapidly than in **E4a**. In particular, mean CPU utilization approaches 100% at ten queries, while the increasing network traffic reflects the additional physical streams introduced by each query. The comparison between **E4a** and **E4b** therefore shows that preserving shared access to the underlying streams provides a substantial scalability benefit even when query executions themselves cannot be reused.

Taken together, the results support the hypothesis associated with Research Question 2: the benefit of shared processing depends on the amount of reusable work present in the workload. The greatest opportunity for reducing duplicated work occurs when both the input streams and the continuous query are shared, as in **E1**. When queries differ but access the same streams, as in **E4a**, stream acquisition can still be shared and the principal scaling cost shifts toward maintaining independent query executions. When both the queries and their physical input streams differ, as in **E4b**, neither form of work can be reused and the complete acquisition-and-processing path must scale with the workload. The substantially earlier degradation observed in **E4b** compared with **E4a** demonstrates the practical importance of stream-level sharing independently of query-result reuse. The evaluation therefore suggests a continuum rather than a binary distinction between centralized and Client-Side Processing: different degrees of stream and query overlap determine which parts of the processing pipeline can be shared.

#### 9.1.3. Processing Completeness and Temporal Ordering

The scalability results also show that latency alone is insufficient to characterize overload in a continuous stream-processing system. In **E1**, Client-Side Processing exhibits increasing latency and event reordering before a substantial loss of run completion is observed. Similarly, in **E4a**, latency increases substantially before the onset of sustained physical-input incompleteness at the highest query counts. In **E4b**, run-level completeness begins to deteriorate at five queries even though mean input completeness remains 95.17%, illustrating that an aggregate completeness value can conceal instability across repetitions.

The temporal-ordering measurements provide an additional indication of this increasing pressure. With independently acquired streams in **E4b**, the out-of-order proportion increases from 20.92% at three queries to 36.92% at five queries and 41.18% at ten queries. Observations first exceed the configured 30 s out-of-order bound at five queries, where maximum lateness reaches 44.34 s, although 99.77% of out-of-order observations still remain within the configured tolerance. By contrast, all out-of-order observations in **E4a** remain within the 30 s bound throughout the evaluated range. These results indicate that scalability should be characterized jointly in terms of latency, processing completeness, and temporal ordering rather than through response latency alone.

At the same time, the correctness measurements help distinguish incomplete physical-input processing from loss within the subsequent query-processing pipeline. Fan-out correctness in **E4a** and retrieval-to-insertion correctness in **E4b** remain 100% for successfully acquired observations. The incomplete runs therefore provide no evidence that Heimdall selectively loses observations during fan-out in **E4a** or between retrieval and RSP insertion in **E4b**. In **E4b**, however, the experiment simultaneously increases load on the replayer, Solid server, stream-acquisition path, and Heimdall service. The measurements therefore do not identify any single component as the sole cause of the missing physical input.

### 9.2. Architectural Limitations

The introduction of Heimdall as a shared stream-processing service introduces several architectural trade-offs. Although the underlying sensor data remains stored in independently controlled Solid Pods, Heimdall becomes a shared processing component that must be trusted to access authorized streams and correctly distribute the corresponding query results. The service also maintains query-registration and subscription state for clients relying on shared query executions. Consequently, the availability of a Heimdall instance can affect multiple clients simultaneously. The current implementation employs single-emission window semantics where once a window is finalized, its state is discarded and subsequently arriving events cannot revise the emitted result. The applications requiring result corrections or retractions for very late events would therefore require retained window state and an update-aware output policy for the emission logic of the windows. Such an update-aware policy could retain the state of a finalized window for a configurable additional period. If a late event belonging to that window arrives during this period, the affected window could be re-evaluated and the previously emitted result could be retracted or replaced by an updated result. This approach would improve completeness for safety-critical applications at the cost of additional state retention, processing overhead, and delayed finality.

The **E4** results also emphasize the importance of the current reuse boundary. Heimdall reuses an execution only when the implemented structural compatibility conditions are satisfied. Queries with partial overlap, shared sub-patterns, or containment relationships therefore execute independently even when some computation could theoretically be reused. Extending the registry with query-containment or multi-query reuse optimization could shift some workloads currently represented by **E4a** toward greater execution sharing. Several functional capabilities also remain outside the scope of the current implementation. Heimdall currently processes newly arriving stream events and does not reconstruct or query historical streams from events already stored in a Solid Pod. Historical access is nevertheless relevant for monitoring applications in which current observations must be interpreted relative to previous behavior. In complementary work, we introduced Janus and Janus-QL [76], which extend the RSP-QL model with declarative windows over persisted RDF event logs and enable historical and live RDF observations to be evaluated within a single query. Janus provides a concrete execution model for historical–live RDF stream processing, but it currently operates as a separate system and has not been integrated into Heimdall. Integrating such historical-window support with Heimdall’s Solid-based access control and shared continuous-query execution therefore remains future work.

Query results are delivered directly to subscribed clients rather than being persistently stored for later retrieval or reuse. The service also does not currently perform semantic reasoning or schema alignment to reconcile heterogeneous vocabularies that may be used by different Pods. Finally, while the prototype authenticates access to Solid resources, the current implementation does not provide fine-grained policy-based authorization for query registration and result sharing. A client receiving a derived result should be authorized not only to access the underlying stream but also to receive the information produced by the corresponding continuous query. Supporting such authorization policies represents an important requirement for deploying shared query-processing services over sensitive personal data. In a broader decentralized ecosystem, multiple independently operated Heimdall instances could potentially discover reusable results, delegate query processing, or cooperate in selecting execution locations. Realizing such a multi-agent architecture would require additional mechanisms for service discovery, trust establishment, authorization propagation, and coordination. This scenario therefore represents a future research direction rather than a capability demonstrated by the current implementation.

### 9.3. Scope and Limitations of the Evaluation

The experimental results should be interpreted within the evaluated workload and deployment configuration. The experiments use accelerometer data from a single DAHCC participant, a controlled family of activity-index queries, one Community Solid Server configuration, one RDF Stream-Processing Engine, and a dedicated local-area network. The stream replay is deterministic at a fixed rate rather than bursty and therefore does not reproduce the original inter-arrival distribution or geographically distributed network conditions. The results consequently characterize the relative behavior of the evaluated architectures under controlled conditions rather than establish performance bounds for arbitrary Solid deployments.

The **E1** multi-client experiment executes all client processes concurrently on one dedicated client host. The resulting measurements intentionally include contention between co-located client processes and therefore represent a contention-aware deployment rather than clients executing on independent physical devices, as would be the case in a decentralized network. The decentralized-client CPU and RSS projections complement these direct measurements by estimating accumulated demand when client resources are independently provisioned, but they should not be interpreted as predictions of latency or saturation under such a deployment.

**E3** isolates query and data heterogeneity using a single client. It therefore establishes that the tested query variants have similar per-query costs but does not by itself characterize scalability under concurrent heterogeneous workloads. **E4** addresses concurrent non-reusable queries within Heimdall but does not repeat the three-architecture comparison from E1. Consequently, **E4** demonstrates how the loss of query-execution and stream-acquisition sharing affects Heimdall itself but does not establish the relative performance ranking of Heimdall, the Notification Aggregator, and Client-Side Processing for every heterogeneous concurrent workload.

The saturation behavior observed in **E4** is also specific to the evaluated hardware, replay rate, query structure, and service configuration. The onset of incomplete processing and the transition into the high-load operating region should therefore be interpreted as experimentally observed behavior for this deployment rather than as a general capacity limit of Heimdall. This distinction is particularly important because the transition is not strictly binary: **E4a** contains isolated incomplete repetitions before sustained degradation appears, while in **E4b** only 9 of 30 runs are complete at five queries despite a mean input completeness of 95.17%.

Furthermore, in **E4b**, increasing the number of queries simultaneously increases load on the replayer, Solid server, network-processing path, and Heimdall service. Consequently, the experiment cannot attribute incomplete physical-input processing to a single component without additional component-isolation experiments, which are outside the scope of this work.

Finally, the evaluation does not consider dynamically changing client populations, bursty or variable-rate streams, geographically distributed Pods, alternative Solid server implementations, different RSP engines, or substantially more complex query shapes. These dimensions remain necessary for evaluating the generality of the observed trade-offs beyond the controlled workload used in this study.

## 10. Conclusions and Future Work

This paper presented Heimdall, an intermediary service for continuous processing of RDF streams in Solid Pods. Heimdall enables clients to register RSP-QL queries and reuses an existing query execution when the supported reuse conditions are satisfied, thereby avoiding duplicated stream acquisition and continuous-query execution. We evaluated Heimdall against Client-Side Processing and the Notifications Aggregator to investigate how the placement and sharing of stream-processing operations affect latency, resource consumption, and network traffic. The results show that sharing both stream acquisition and query execution allows Heimdall to maintain stable latency as the number of clients requesting the same computation increases, while architectures that retain more processing at each client exhibit greater growth in latency, accumulated resource consumption, and network traffic. The latency decomposition further shows that Heimdall introduces additional one-time initialization operations, while the Heimdall-specific query-management operations contribute comparatively little latency relative to recurring stream retrieval and query evaluation. Under the evaluated single-client heterogeneous workloads, varying the query and input data has only a limited effect on the latency, resource consumption, and network behavior of each architecture.

The experiments with concurrent non-reusable queries further show that the scalability of Heimdall depends on which parts of the processing pipeline can still be shared. When independent queries operate over the same physical streams, stream acquisition remains shared and physical-input processing remains highly complete through 30 concurrent query executions, with mean completeness remaining at or above 96.42% over this range. Sustained degradation appears at 35 queries, although latency and resource consumption increase substantially before this point. When both the queries and their physical streams differ, the opportunity for sharing is further reduced. All retained repetitions remain complete through three concurrent queries, while degradation appears at five queries and becomes substantial at seven and ten queries. In both configurations, successfully retrieved observations continue to be inserted correctly into the corresponding query executions. These findings show that the scalability benefit of shared stream processing is determined by the degree of reusable work, as sharing stream acquisition reduces duplicated access to the decentralized data source, while additionally sharing query execution reduces repeated computation and result-delivery overhead.

Future work will extend Heimdall beyond the current structural query-reuse mechanism by investigating query-containment-based and partial query reuse for RSP-QL workloads. We also plan to integrate historical and live stream processing within a unified execution model, investigate persistent storage and reuse of derived query results, and introduce policy-aware authorization for query registration and result sharing. Further evaluation will consider variable and bursty stream rates, more diverse and complex query workloads, geographically distributed Solid Pods, alternative Solid server and RSP-engine implementations, and coordination between multiple Heimdall instances for distributed query execution and result reuse. We also plan to investigate multi-agent deployments in which independently operated Heimdall instances coordinate query execution and result reuse, potentially using LLM-assisted agents for high-level query planning and orchestration, service discovery, and execution-location selection while retaining deterministic RSP engines for the actual stream processing.

## Figures and Tables

**Table 1:** Run success rate and failures under increasing concurrent client load. Each configuration was attempted 30 times. Failed runs are included in the success rate but excluded from latency summaries.

Clients	Heimdall		Notification Aggregator		Client-Side Processing	
	Success (%)	Failed	Success (%)	Failed	Success (%)	Failed
1	100.0	0	100.0	0	100.0	0
3	100.0	0	100.0	0	100.0	0
5	100.0	0	100.0	0	100.0	0
7	100.0	0	100.0	0	100.0	0
10	100.0	0	100.0	0	100.0	0
11	100.0	0	100.0	0	93.3	2
12	100.0	0	100.0	0	40.0	18

**Table 2:** Latency decomposition for the three stream-processing approaches with one client. Latency values are reported in milliseconds as mean ± standard deviation on the first line and median [$Q_{1}$, $Q_{3}$] on the second line for recurring operations. Statistics are calculated across the 30 retained experimental repetitions (iterations 04–33). A dash indicates that the operation is not applicable, while n.m. indicates that it was not separately measured.

Operation	Client-Side	Notifications Aggregator	Heimdall
*One-time initialization*			
Service discovery	–	25.65±2.34.	21.33±4.53
Stream discovery	18.86±3.82	25.96±5.81	21.45±4.33
Query reuse check	–	–	0.19±0.02
Service authentication	–	35.4±6.5.	28.45±3.4
Query registration	–	–	0.27±0.04
Stream subscription	37.64±5.79	0.24±0.06	85.85±9.16
WebSocket query message	–	–	0.64±0.33
*Recurring operations*			
Event retrieval	14.22 ± 0.33 14.25 [14.05, 14.34]	7.20 ± 0.12 7.20 [7.14, 7.30]	21.68 ± 2.47 21.99 [19.67, 23.32]
Parsing and timestamp extraction	0.25 ± 0.02 0.25 [0.24, 0.26]	0.45 ± 0.02 0.45 [0.44, 0.46]	1.34 ± 0.04 1.33 [1.31, 1.36]
Insertion into RSP engine	0.27 ± 0.01 0.27 [0.26, 0.28]	0.60 ± 0.03 0.60 [0.58, 0.62]	0.55 ± 0.03 0.55 [0.53, 0.57]
Query evaluation	76.54 ± 12.42 77.12 [73.59, 81.16]	120.71 ± 15.84 109.75 [107.63, 137.86]	51.52 ± 14.77 45.65 [43.72, 46.97]
Result dispatch	–	–	0.07 ± 0.01 0.06 [0.06, 0.07]

**Table 3:** First-result latency across the E3 query and data heterogeneity scenarios. Values are reported in milliseconds as mean ± standard deviation across 30 retained repetitions.

Workload Scenario	Heimdall (ms)	Notification Aggregator (ms)	Client-Side Processing (ms)
Same query/same data	160.45±30.29	204.31±11.67	156.85±14.41
Different queries/same data	157.54±11.55	205.53±8.19	164.82±12.39
Different queries/different data	158.71±12.59	206.27±10.36	162.59±12.66

**Table 4:** Client- and service-side resource consumption across the E3 query and data heterogeneity scenarios. Values are reported as mean ± standard deviation across 30 retained repetitions. A dash indicates that no intermediary service is present.

Scenario	Architecture	Client CPU (%)	Client RSS (MB)	Service CPU (%)	Service RSS (MB)
Same query/same data	Heimdall	2.67±0.20	230.55±2.74	58.21±1.23	216.06±6.76
	Notification Aggregator	10.41±0.22	345.70±4.63	3.95±0.15	67.75±0.39
	Client-Side Processing	23.85±3.86	357.91±6.15	–	–
Different query/same data	Heimdall	2.69±0.12	231.28±1.46	57.98±0.61	216.69±3.83
	Notification Aggregator	10.72±1.99	345.83±2.59	3.94±0.08	67.82±0.26
	Client-Side Processing	22.88±1.39	355.40±3.13	–	–
Different query/different data	Heimdall	2.66±0.14	230.95±1.44	58.17±0.61	215.73±3.24
	Notification Aggregator	10.78±2.16	344.86±2.95	3.95±0.10	67.76±0.30
	Client-Side Processing	22.83±1.07	354.92±2.94	–	–

**Table 5:** Network traffic across the E3 query and data heterogeneity scenarios. Values represent mean bandwidth in Mbps across the retained repetitions. A dash indicates that no intermediary service is present.

Scenario	Architecture	Client RX	Solid TX	Service TX
Same query/same data	Heimdall	0.026	0.508	0.084
	Notification Aggregator	0.125	0.504	0.176
	Client-Side Processing	0.780	0.857	–
Different query/same data	Heimdall	0.026	0.508	0.084
	Notification Aggregator	0.125	0.504	0.176
	Client-Side Processing	0.780	0.857	–
Different query/different data	Heimdall	0.027	0.508	0.084
	Notification Aggregator	0.125	0.504	0.176
	Client-Side Processing	0.782	0.858	–

**Table 6:** Physical-stream processing completeness and fan-out correctness under an increasing number of independent query executions over shared physical streams. Each configuration contains 30 retained repetitions.

Queries	Complete Runs (%)	Mean Input Completeness (%)	Runs with Correct Fan-Out (%)
1	100.0	100.00	100
10	90.0	96.42	100
20	100.0	100.00	100
25	100.0	100.00	100
30	93.3	97.65	100
35	0.0	78.50	100
40	13.3	86.75	100

**Table 7:** Temporal ordering behavior under an increasing number of independent query executions over shared physical streams. Out-of-order proportions are calculated over observations successfully classified by the RSP engine, while lateness statistics are calculated only for observations classified as out of order.

Queries	Out of Order (%)	Mean Lateness (s)	Max Lateness (s)	Within 30 s Bound (%)
1	0.02	0.25±0.00	0.25	100.00
10	0.71	0.33±0.14	0.75	100.00
20	3.05	0.71±0.49	2.76	100.00
25	2.09	0.39±0.22	1.50	100.00
30	3.70	0.58±0.39	3.26	100.00
35	5.80	3.55±3.13	15.54	100.00
40	6.55	3.12±3.21	15.54	100.00

**Table 8:** Processing completeness and retrieval-to-insertion correctness under an increasing number of independent queries over different physical streams. Each configuration contains 30 retained repetitions.

Queries	Complete Runs (%)	Mean Input Completeness (%)	Correct Retrieval-to-Insertion (%)
1	100.0	100.00	100
3	100.0	100.00	100
5	30.0	95.17	100
7	0.0	74.59	100
10	3.3	56.34	100

**Table 9:** Temporal ordering behavior under an increasing number of independent queries over different physical streams. Out-of-order proportions are calculated over observations successfully classified by the RSP engine, while lateness statistics are calculated only for observations classified as out of order.

Queries	Out of Order (%)	Mean Lateness (s)	Max Lateness (s)	Within 30 s Bound (%)
1	0.00	–	–	–
3	20.92	2.45±2.59	21.54	100.00
5	36.92	5.44±5.48	44.34	99.77
7	40.58	6.89±6.99	53.63	98.95
10	41.18	8.31±8.54	53.08	97.10

## Data Availability

The dataset used in this study is publicly available from the Data Analytics for Health and Connected Care (DAHCC) project at https://dahcc.idlab.ugent.be/dataset.html (accessed on 9 September 2026).

## Abbreviations

The following abbreviations are used in this manuscript:

AI	Artificial Intelligence
CQ	Continuous Query
CQL	Continuous Query Language
CSS	Community Solid Server
GDPR	General Data Protection Regulation
HTTP	Hypertext Transfer Protocol
IRI	Internationalized Resource Identifier
IoT	Internet of Things
LDES	Linked Data Event Streams
LDP	Linked Data Platform
LDESTS	Linked Data Event Time Series
LWS	Linked Web Storage
OIDC	OpenID Connect
PROTEGO	Personalized alaRming and cOntextualized dispaTching through lifEstyle monitorinG
R2R	Relation-to-Relation
R2S	Relation-to-Stream
RDF	Resource Description Framework
RSP	RDF Stream Processing
RSP-QL	RDF Stream Processing Query Language
S2R	Stream-to-Relation
SDS	RSP-QL Dataset
SPARQL	SPARQL Protocol and RDF Query Language
URI	Uniform Resource Identifier
URL	Uniform Resource Locator
W3C	World Wide Web Consortium
